# PALB2 deficiency may sensitize H3K27M-mutant pediatric HGG cells to BMN673/talazoparib

**DOI:** 10.3389/fonc.2025.1589396

**Published:** 2025-06-30

**Authors:** Xiaowen Guan, Xinke Xu, Xiaolan Mo, Houliang Deng

**Affiliations:** ^1^ School of Life Sciences, Zhengzhou University, Zhengzhou, Henan, China; ^2^ Department of Neurosurgery, Guangzhou Women and Children's Medical Center, Guangzhou Medical University, Guangzhou, Guangdong, China; ^3^ Department of Pharmacy, Guangzhou Women and Children's Medical Center, Guangzhou Medical University, Guangzhou, Guangdong, China

**Keywords:** H3K27M-mutant pHGG, BMN673, PARP1 trapping, DNA repair, PALB2

## Abstract

**Backgroud:**

Pediatric high-grade glioma (pHGG) with the histone H3 Lys27Met substitution (H3K27M) is a devastating disease with a high mortality rate in children and adolescents (from birth to 19 years of age). No effective treatments have been developed for this tumor type. Thus, a better understanding of the underlying complex mechanisms and identify more potential drugs targeting H3K27M-mutant pHGG are urgently needed.

**Methods:**

In the current study, we established pHGG cell models harboring H3K27M by transfecting two pHGG cell lines, SF188 and Res259, with the H3K27M mutant and H3 wild-type (WT) plasmids and then performed drugs screening. Then we employed an EJ5 reporter assay to measure nonhomologous end joining (NHEJ) activity. Western blotting was used to analyze DNA damage markers (γ-H2AX and PLK1), and cell cycle progression was assessed. Additionally, we utilized whole-exome sequencing and CRISPR/Cas9 genome editing to generate Res259 cell lines with stable deficiencies in ARID1A, P53, or PALB2, followed by viability assays to evaluate drug sensitivity.

**Results:**

Notably, BMN673 (talazoparib) was identified as a synthetic lethal hit in the H3K27M-mutant SF188 cell model. However, BMN673 did not affect the constructed H3K27M-mutant Res259 cells. Moreover, we showed that the H3K27M mutation induced an aberrant increase in NHEJ activity. Furthermore, BMN673 treatment increased the protein levels of γ-H2AX and PLK1, induced cell cycle arrest, and promoted PARP1 trapping in H3K27M-mutant SF188 cells. In addition, the results of a series of viability assays revealed that the H3K27M mutation combined with PALB2 deficiency sensitized H3K27M-mutant Res259 cells to BMN673. However, deficiencies in ARID1A or P53 did not produce similar effects.

**Conclusion:**

Overall, our results may provide some reference value for further study of the effects of BMN673 and PALB2 deficiency in the treatment of H3K27M-mutant pHGG.

## Introduction

Brain and central nervous system tumors are the leading causes of cancer-related death and the second most common type of cancer in children and adolescents (1). Among the various types of brain tumors, pediatric high-grade gliomas (pHGGs) are the deadliest. Even with combinations of the most advanced treatments, the survival time of patients with this disease is only approximately 9–12 months (2). The poor prognosis of pHGGs and the lack of effective targeted therapies remain major problems. Thus, understanding the molecular mechanisms of pHGGs and identifying targets for its treatment are highly important.

Genetic analyses have revealed that somatic mutations such as the Lys27Met substitution in histone H3.3 (H3.3K27M) or histone H3.1 (H3.1K27M) frequently occur in pHGGs (3, 4). Main type H3K27M-mutant pHGGs are diffuse midline gliomas (DMGs) with H3K27 alterations, which are found primarily in the brainstem (pons), thalamus and spinal cord. Other types of pHGGs include diffuse hemispheric gliomas (DHG) with H3.3 G34R/V mutations, infant HGG (which often have gene fusions) and pHGGs with receptor tyrosine kinase (RTK) type alterations or IDH1/2 mutations (5–8), respectively. These types of pHGGs are well described in the World Health Organization (WHO) Classification of Central Nervous System Tumors (5th edition, 2021). Histones with the H3K27M mutation has been reported to bind the SET domain of EZH2 (enhancer of zeste homolog 2), a core component of polycomb repressive complex 2 (PRC2), resulting in global loss of H3K27 di- and trimethylation (H3K27me2/3) (9, 10) and DNA hypomethylation. Specially, the global reduction in H3K27me2/3 levels and DNA hypomethylation may cooperate to drive gliomagenesis (11, 12). Despite the prevalence of the H3K27M genetic mutation, a rational therapeutic approach to target cancers with this mutation has not been developed. To date, several potential targeted drugs have been identified in *in vitro* studies (13–16), but none of them are being used in the clinic. Therefore, to further explore more potential drugs for H3K27M-mutant pHGGs, we screened a panel of drugs or inhibitors to determine their efficacy against two pHGG cell models generated in our lab that harbor the H3K27M mutation(H3K27M-mutant SF188 and H3K27M-mutant Res259) (17), and the results revealed that PARP1 inhibitor BMN673/talazoparib may selectively act on H3K27M-mutant SF188 cells.

PARP1 is a crucial protein for the sensing of DNA double-strand breaks (DSBs) and can be activated by unligated Okazaki fragments during the replication phase (18, 19). Several PARP1 inhibitors, such as olaparib, niraparib, and BMN673, have been developed. The advent of PARP inhibitors has changed the management of patients with ovarian cancer, and their effectiveness has been demonstrated, especially in homologous recombination (HR)-deficient tumors (20). These PARP inhibitors have similar mechanisms, including reducing PARP1 activity for lesion repair and PARP1 trapping, and some studies have demonstrated that PARP1 trapping exerts stronger cytotoxic effects than reducing PARP1 activity (21, 22). More specifically, the PARP1-DNA-trapping complex poses a barrier that impedes essential cellular processes such as DNA replication, resulting in replication fork collapse and eventually in cell death (23–25).

In this study, it was unexpectedly found that the PARP1 inhibitor BMN673 had different effects on the H3K27M-mutant SF188 cells and H3K27M-mutant Res259 cells that we generated. Notably, we demonstrated that treatment with BMN673 could induce cell cycle arrest, promote PARP1 trapping and drive an aberrant increase in NHEJ repair activity only in H3K27M-mutant SF188 cells significantly. Importantly, PALB2 deficiency was found to increase the BMN673 sensitivity of H3K27M-mutant Res259 cells, which are initially resistant to BMN673, indicating that there may be a complex relationship between PALB2, H3K27M, and the drug BMN673. Our preliminary findings not only provide novel directions for further in-depth research on more relevant topics, but also offer preliminary experimental evidence showing that BMN673 treatment may be potential approaches for therapeutic interventions against pHGGs with the H3K27M mutation.

## Materials and methods

### Generation of stable H3K27M-mutant cell lines

The pediatric glioma cell line SF188 was obtained from Dr. Daphne Haas-Kogan (Dana Farber Cancer Institute), and the pediatric glioma cell line Res259 was obtained from Dr. Michael Bobola (University of Washington). Both SF188 and Res259 cells lines are H3K27M (H3.1 and H3.3) wild type. These two cell lines were used to generate stable H3 WT cell lines and H3K27M-mutant cell lines (**Supplementary Figure S1**). Briefly, in accordance with the protocol of the QuikChange Lightning Site Directed Mutagenesis Kit (Agilent Technologies, USA), the H3K27M (c.83A>T) mutation was introduced into the PCMV6-H3F3A (C-terminal myc-DDK-tagged) plasmid (OriGene Technologies, USA). After sequencing and confirmation of the correct mutant plasmid, the H3K27M mutant plasmid or H3 WT plasmid (PCMV6-H3F3A) was transfected separately into SF188 and Res259 cells with Lipofectamine 2000 transfection reagent (Invitrogen, USA). For the selection of stably transfected clones, we used medium supplemented with 1 mg/mL G418 (Thermo Fisher Scientific, USA) and then performed Western blotting to detect the H3K27M mutation with an anti-H3K27M antibody (Merck Millipore, Germany) to obtain the correct clones. All cells were cultured in DMEM supplemented with 10% fetal bovine serum (Thermo Fisher Scientific, USA), maintained in a humidified incubator set to 5% CO2 and 37°C and confirmed to be negative for mycoplasma contamination by using the MycoAlert Mycoplasma Detection Kit (Lonza, Switzerland).

### Drug library screening and cell viability assays

The compound library (L1900) (Selleck Chemicals, USA) containing 128 small molecule inhibitors was a gift from Prof. Shim’s laboratory at the University of Macau. All the inhibitors were screened in a 384-well plate. The concentration of each inhibitor ranged from 14 nM to 30 nM, and the assay format was interplate titration. SF188 and Res259 cells (H3 WT and H3K27M mutation) were seeded into 384-well plates at 2000 cells per well. After 72 h of incubation, the viability of the cells was measured. The cells were treated with 10% alamarBlue solution (Sigma–Aldrich, USA) for 3 h, and the signals were measured with a SpectraMax-M5 plate reader (Molecular Devices) at an excitation wavelength of 560 nm and an emission wavelength of 590 nm (ex560/em590). After the signal in each well was measured, the half-maximal inhibitory concentration (IC50) values of each inhibitor were calculated with GraphPad Prism 6.0 (version 6, USA). These screening assays were performed two times, and the average IC50 values were recorded to calculate the selectivity index (SI) values with the following equation: IC50 H3WT cells/IC50 H3K27M-mutant cells. An SI > 2 indicated that treatment with the compound may be a synthetic lethal hit.

### Cell viability assays with the selected candidate inhibitors and previously reported inhibitors

Cells were seeded into 96-well culture plates at 5000 cells per well. BMN673, JQ1, AZ960, LBH589, sorafenib, and EPZ6438 (all purchased from Selleck Chemicals; concentrations of 0.01 µM, 0.05 µM, 0.1 µM, 0.5 µM, 1 µM, 5 µM, 10 µM, 50 µM, and 100 µM) or 0.01% DMSO were added. BMN673 was identified from the drug library in our study screening as a candidate drug effective against the H3K27M-mutant SF188 cells that we generated. JQ1 (a BET inhibitor) and AZ960 (a JAK2 inhibitor) were identified as candidate drugs effective against the H3K27M-mutant Res259 cells that we generated. LBH589 (26), sorafenib (27, 28), and EPZ6438 (15) are drugs reported to be potentially effective against gliomas. After treatment for 72 h, cell viability was determined via a CCK-8 assay (Beyotime Biotechnology, China). The absorbance was subsequently measured using a multimode plate reader (Tecan, Infinite 200 PRO, Switzerland). We used Prism 6 software to calculate the IC50 values in each group.

### Generation of EJ5 reporter cell lines

H3K27M-mutant cell lines and H3WT Res259 cell lines were transfected with PimEJ5GFP (Addgene, #44026) using Lipofectamine 2000 transfection reagent (Invitrogen, USA). For the selection of stably transfected clones, we used medium supplemented with 1 μg/mL puromycin (Thermo Fisher, USA) and evaluated GFP fluorescence to select the EJ5 reporter cell clones. The correct clones were further verified via Sanger sequencing.

### EJ5 reporter assay

H3K27M-mutant cell lines and H3 WT cell lines containing a stably integrated copy of the EJ5 reporter were used to assess the repair of I-SceI-induced DSBs via the NHEJ repair pathway (29, 30). The indicated EJ5 reporter cell lines were transfected with two plasmids, namely, the I-SceI expression vector pCBASce (Addgene #26477) and the DsRed expression vector (Addgene #54493), 48 h later, the percentage of GFP-positive cells among DsRed-positive cells was evaluated by imaging with a Carl Zeiss LSM 710 confocal fluorescence microscope and quantified via FACS on a BD Accuri™ C6 flow cytometer (BD Biosciences, USA) using BD Accuri™ software.

### Subcellular protein extraction

Subcellular protein fractions were collected with a subcellular fractionation kit (Thermo Fisher Scientific, USA). In brief, the cells were harvested, centrifuged at 2500 rpm for 5 min, washed with cold PBS, and centrifuged for 5 min at 2500 rpm at 4°C. CEB buffer was added to the cells, which were gently mixed well, incubated on ice for 10 min, and then centrifuged for 4 min at 500 × g. The supernatant was collected as the cytoplasmic protein fraction. MEB buffer was then added, the pellet was resuspended, and the mixture was incubated on ice for 10 min and centrifuged for 5 min at 3000 × g. The supernatant was collected as the membrane protein fraction. Next, NEB buffer was used to extract the soluble nuclear proteins. NEB buffer was mixed with the pellet, and after incubation for 30 min on ice and centrifugation for 5 min at 5000 × g, the supernatant was collected as the soluble nuclear protein fraction. Nuclease and CaCl_2_ were added to NEB buffer at room temperature, and this buffer was added to the pellet, which was mixed well and vortexed for 15 sec. Then, the mixture was placed in a 37°C heat block for 5 min and centrifuged for 5 min at 16000 × g, after which the supernatant containing the chromatin-bound proteins was collected. Each protein sample was subsequently measured via BCA assays and boiled for Western blot analysis.

### Acid extraction of histones

Approximately 1×10^7^ cells were collected and washed twice with PBS. The cell pellet was resuspended in 1 mL of prechilled hypotonic lysis buffer and transferred to a 1.5 mL centrifuge tube. The mixture was incubated with rotation at 4°C for 30 minutes to induce hypotonic swelling and mechanical shearing during rotation. The mixture was centrifuged at 10,000 × g for 10 minutes at 4°C, and the supernatant was discarded. The nuclear pellet was resuspended thoroughly in 400 µL of 0.2 M H_2_SO_4_ and then incubated with rotation at 4°C for at least 30 minutes. Then, the samples were centrifuged at 16,000 × g for 10 minutes at 4°C to remove nuclear debris. The supernatant containing the histones was transferred to a new 1.5 mL centrifuge tube. TCA (trichloroacetic acid) was added to the histone mixture, which was then placed on ice for at least 30 minutes. The mixture was centrifuged at 16,000 × g for 10 minutes at 4°C to pellet the histones, and the supernatant was discarded. The histone pellet was washed with prechilled acetone without disturbing the pellet. After centrifugation at 16,000 × g for 5 minutes at 4°C, the supernatant was discarded, and the histone pellet was air dried for 20 minutes. The histone pellet was dissolved in an appropriate amount of ddH_2_O. Finally, the protein concentration was determined via the BCA method.

### Western blot analysis

The cells were lysed in RIPA lysis buffer (Invitrogen, USA) supplemented with 1% (v/v) phosphatase inhibitor cocktail (Sigma–Aldrich, USA). A total of 50~60 µg of protein was separated via SDS–PAGE, after which the proteins were transferred to nitrocellulose membranes (Pall Corporation, USA) with an electrophoretic transfer apparatus (Bio-Rad, USA). After transfer, the membrane was blocked with 5% nonfat milk for 2–3 h and then incubated with the following antibodies at 4°C overnight: rabbit polyclonal anti-PARP1 (1:1000 dilution, Abcam, UK), rabbit monoclonal anti-GAPDH (1:2500 dilution, Cell Signaling Technology, USA), rabbit monoclonal anti-γ-H2AX (1:10000 dilution, Cell Signaling Technology, USA), mouse monoclonal anti-p53 (1:2000 dilution, Abcam, UK), rabbit monoclonal anti-ARID1A (1:1000 dilution, Abcam, UK) and rabbit monoclonal anti-PALB2 (1:1000 dilution, Cell Signaling Technology, USA). After being washed three times with PBST, the membrane was incubated for 2 h with horseradish peroxidase-conjugated anti-rabbit/mouse secondary antibodies (1:5000 dilution, Jackson Immune Research, USA) and washed three times with PBST, after which signals were detected by using ECL solution (Thermo Fisher Scientific, USA).

### Cell cycle assay

The cell cycle distribution was analyzed with a standard flow cytometry protocol including propidium iodide (PI) staining. In brief, the cells were washed with ice-cold PBS and then fixed with 70% ethanol overnight at −20°C. The cells were then stained with PI (Beyotime Biotechnology, China) at room temperature for at least 30 min, analyzed immediately with a BD Accuri C6 flow cytometer (BD Biosciences, USA) and quantified with BD Accuri™ software.

### Generation of ARID1A-deficient and P53-deficient cell lines

The P53 CRISPR–Cas9 knockout plasmid (sc-419469, Santa Cruz Biotechnology, USA) or ARID1A CRISPR–Cas9 knockout plasmid (sc-400116, Santa Cruz Biotechnology, USA), both comprising a pool of three guide RNAs (gRNAs), and the corresponding homology-directed DNA repair (HDR) plasmid containing an HDR template with a dual selection marker (red fluorescent protein (RFP) and puromycin resistance gene) were used. H3K27M-mutant Res259 cells and H3 WT Res259 cells were transfected separately with the P53 knockout plasmid and ARID1A knockout plasmid via Lipofectamine 3000 (Invitrogen, USA). After 48 h of transfection, 1 μg/mL puromycin was added to the medium, and RFP fluorescence was used to select the positive clones. The expression of P53 and ARID1A was further verified by Western blotting.

### Generation of PALB2-deficient cell lines

To knock out PALB2, two pairs of single guide RNAs (sgRNAs) targeting exon 2 (5’-GGGCCTGAGTCCTTTAACCC-3’) and exon 4 (5’- GTCTTTCAAATGAGCAAGTT-GGG-3’) of PALB2 were designed via an online tool (https://zlab.bio/guidedesign-resources). The sgRNA oligos were subsequently cloned and inserted into pSpCas9(BB)-2A-Puro (PX459) V2.0, which was purchased from Addgene (#62988). H3K27M-mutant Res259 cells and H3 WT Res259 cells were transfected for 48 h with plasmids containing the sgRNAs via Lipofectamine 2000 (Invitrogen, USA), and puromycin (1 µg/mL) was then added to the culture medium to select stably transfected clones. PALB2 expression was further verified by Sanger sequencing and Western blotting.

### Statistical analysis

Data analyses were conducted via GraphPad Prism software (version 6, USA). Student’s t test was used to compare values between two groups, and one-way ANOVA was used when comparing three or more groups. The data are presented as the mean ± standard deviation (SD) from three biological replicates. Differences were considered statistically significant at *p* < 0.05.

## Results

### The PARP1 inhibitor BMN673 had different effects on the two constructed H3K27M-mutant pHGG cell lines

**Figure 1:**
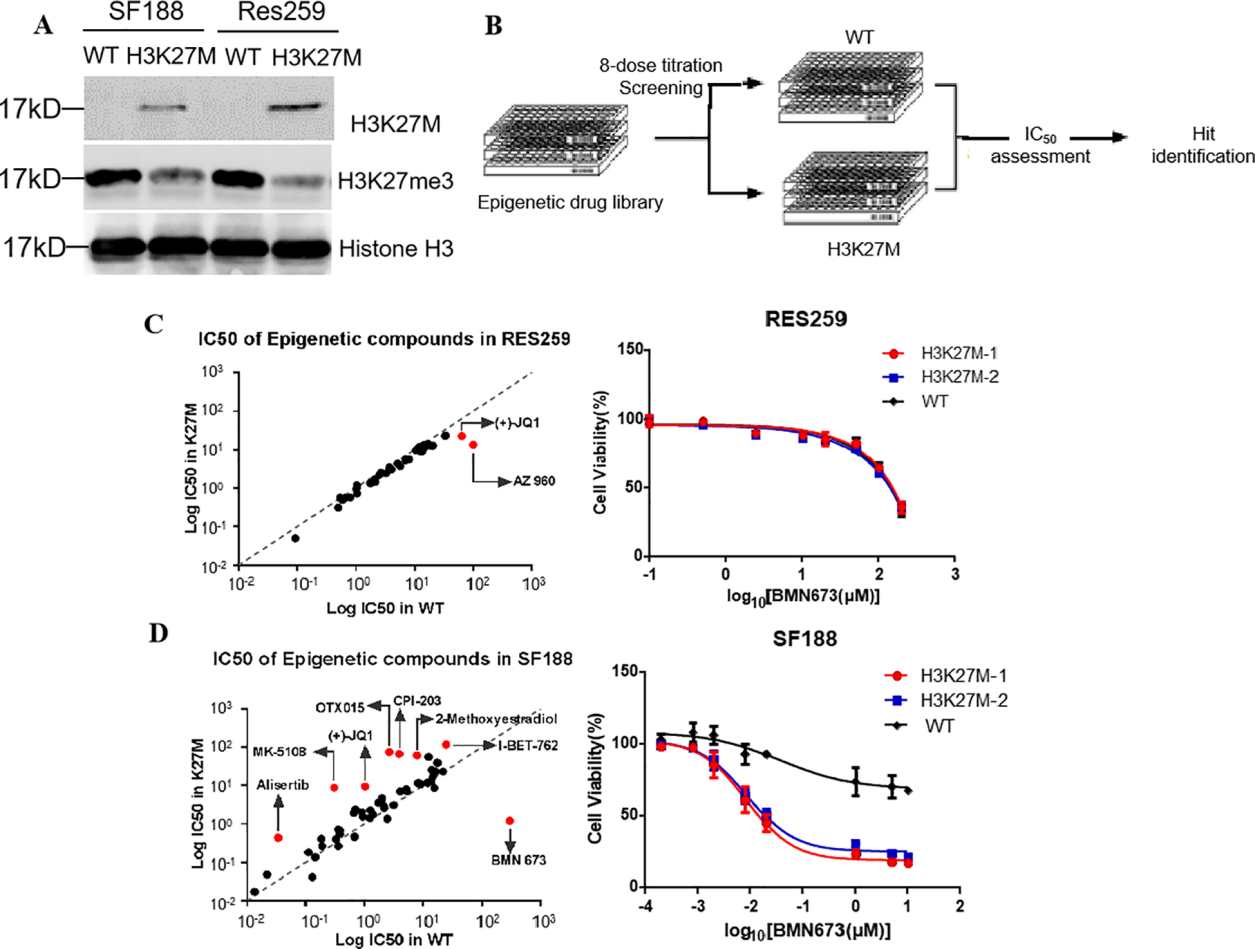
Screening the potential drugs using the two constructed H3K27M-mutant pHGG cell lines. **(A)** The SF188 and Res259 cell lines were stably transfected with constructs encoding H3 WT form or a similarly H3K27M. Acid-extracted histones were subjected to Western blot analysis with the indicated antibodies. **(B)** Schematic illustration of the process for screening potential drugs in drug library. **(C, D)** Left panel: Log10(IC50) plots of the screening data. The classes of compounds that showed candidate drugs in the H3K27M-mutant SF188 and Res259 cell lines are marked on the plots. The average IC50 values from three independent screens are plotted. Right panel: Dose–response curves for the indicated cells treated with the BMN673. The data are presented as the mean ± SD from three independent experiments.

To identify drugs exhibiting synthetic lethality with the H3K27M mutation, an compound library of 128 small molecules targeting most of the druggable human epigenetic targets was first screened in the cells. These cells were denoted H3K27M-mutant SF188 cells, WT SF188 cells, H3K27M-mutant Res259 cells and WT Res259 cells, respectively (**Figure 1A**). Consistent with previous studies (3, 10, 11), the levels of H3K27 trimethylation (H3K27me3) in the two H3K27M-mutant cell lines were significantly lower than those in the corresponding WT cell lines (**Figure 1A**). Next, we screened a library of 128 small molecules that target most druggable human proteins, with the small molecules arrayed in 384-well plates in an eight-dose, interplate titration format (**Figure 1B**). The IC50 of each drug in each cell line was calculated. With (IC50 in H3K27M cells/IC50 in WT cells) >2 as the threshold criterion, we identified one candidate drug, BMN673 (talazoparib, a PARP1 inhibitor), for the treatment of the H3K27M-mutant SF188 cell line and two candidate drugs, JQ1 (a BET inhibitor) and AZ960 (a JAK2 inhibitor), for the treatment of the H3K27M-mutant Res259 cell line (**Figures 1C, D**). Interestingly, BMN673 was selective only for H3K27M-mutant SF188 cells (IC50: 0.07 μM, **Figure 1D**), as it was not effective against H3K27M-mutant Res259 cells (IC50>1000 μM, **Figure 1C**).

Furthermore, we compared the lethality of BMN673 with that of the other selected candidate inhibitors: a BET inhibitor (JQ1) (**Supplementary Figure S2A**), a JAK2 inhibitor (AZ960) (**Supplementary Figure S2B**), and three inhibitors with reported activity against H3K27M-mutant gliomas, namely, EPZ6438 (an EZH2 inhibitor) (**Supplementary Figure S3A**), sorafenib (a multikinase inhibitor) (**Supplementary Figure S3B**), and LBH589 (an HDAC inhibitor) (**Supplementary Figure S3C**). However, the results revealed that JQ1, AZ960, EPZ6438 and sorafenib did not have obvious lethality effects on the two constructed H3K27M-mutant cell lines, and only LBH589 showed selectivity for H3K27M-mutant SF188 cells compared with WT SF188 cells (0.003 μM vs. 0.023 μM, **Supplementary Figure S3C**). Notably, LBH589, which can be called Panobinostat, is a potent and orally active nonselective HDAC inhibitor and has antineoplastic activities (31). It has been reported to be used for the study of refractory or relapsed multiple myeloma (32).

### H3K27M mutation induced a significant increase in NHEJ activity in H3K27M-mutant SF188 cells

**Figure 2:**
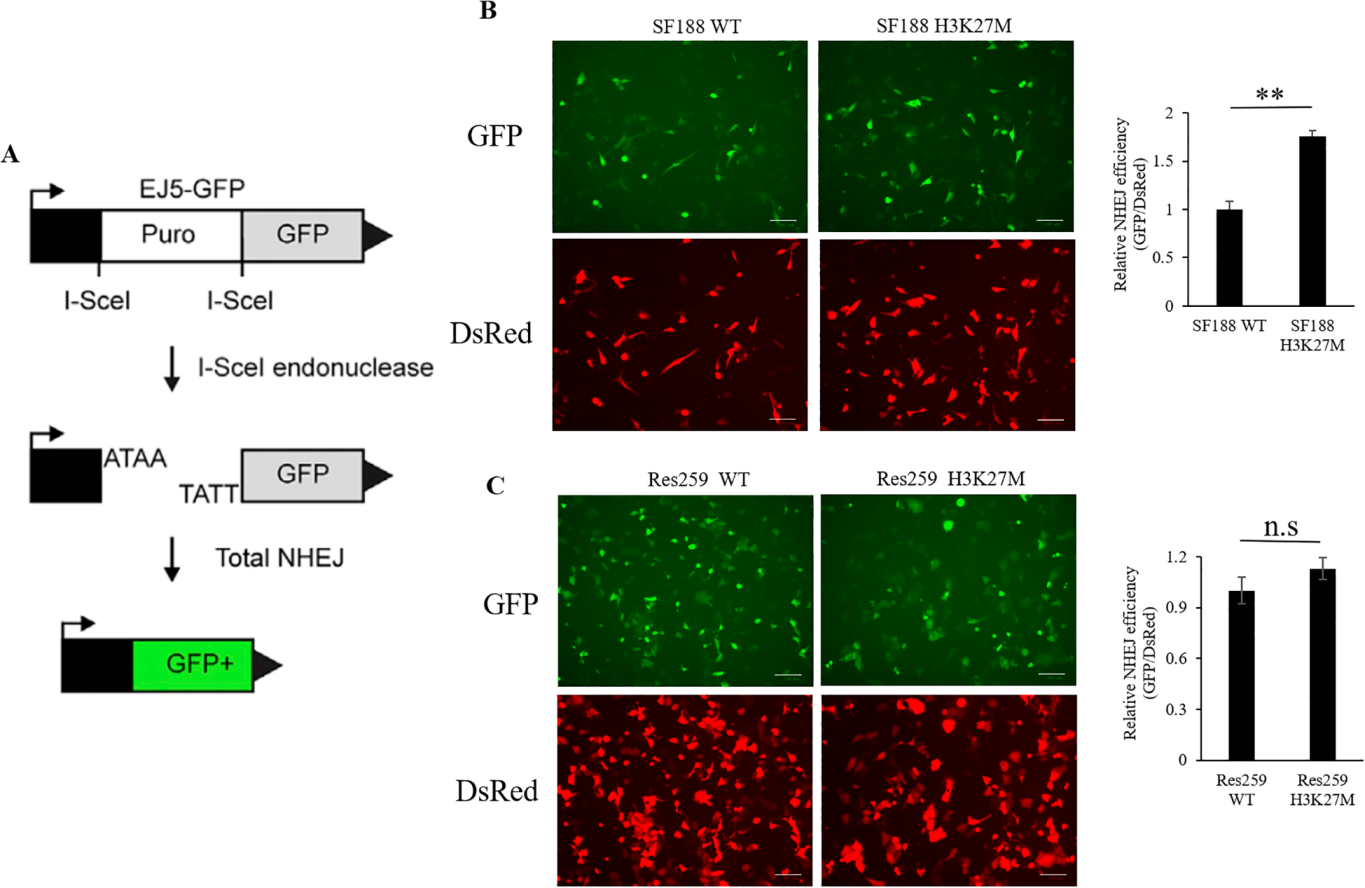
Analysis of NHEJ activity via the EJ5 reporter assay in SF188 and Res259 cells stably expressing the H3 WT or H3K27M mutation. **(A)** Schematic representation of the EJ5 reporter for NHEJ repair. EJ5-GFP is shown along with two classes of NHEJ repair products that can restore a GFP expression cassette. In the starting construct, the GFP gene is inactive. GFP expression is only activated when I-Sce1-induced DSBs are successfully repaired via NHEJ. **(B)** H3K27M-mutant SF188 cells and WT SF188 cells containing the reporter cassette were transfected with the I-SceI and DsRed plasmids. Left panel: Immunofluorescence images of GFP-positive cells and DsRed-positive cells (scale bars, 100 µm). Right panel: NHEJ activity in H3K27M-mutant SF188 cells and WT SF188 cells. **(C)** H3K27M-mutant Res259 cells and WT Res259 cells containing reporter cassettes were transfected with I-SceI and DsRed plasmids. Left panel: Immunofluorescence images of GFP-positive cells and DsRed-positive cells (scale bars, 100 µm). Right panel: NHEJ activity in H3K27M-mutant Res259 cells and WT Res259 cells. NHEJ activity was determined by normalizing the percentage of eGFP-positive cells to the percentage of DsRed-positive cells. The data are shown as the mean ± SD from three independent experiments. Student’s t test was used to calculate *p* values. **p < 0.01 versus the corresponding control.

Previous study demonstrated that the sensitivity of several PARP1 inhibitors is closely correlated with DNA DSB repair pathways, especially the NHEJ pathway (33–35). Next, to assess whether the efficiency of BMN673 in H3K27M-mutant SF188 cells is related to NHEJ activity, we performed an EJ5 reporter assay (29) to study the NHEJ repair activity in the H3K27M-mutant cells that we generated (**Figure 2A**). As shown in **Figures 2B, C**, NHEJ activity was significantly greater in H3K27M-mutant SF188 cells than in WT SF188 cells (*p* < 0.01). Although NHEJ activity was increased in H3K27M-mutant Res259 cells, the increase trend was not significant (*p* > 0.05; p=0.0899). Taken together, these findings indicate that the H3K27M mutation drives aberrant NHEJ activity in H3K27M-mutant SF188 cells, resulting in a significant increase in NHEJ activity, which is consistent with the findings of a previous study (33).

### BMN673 treatment increased the level of γ-H2AX and downregulated PLK1 in H3K27M-mutant SF188 cells

**Figure 3:**
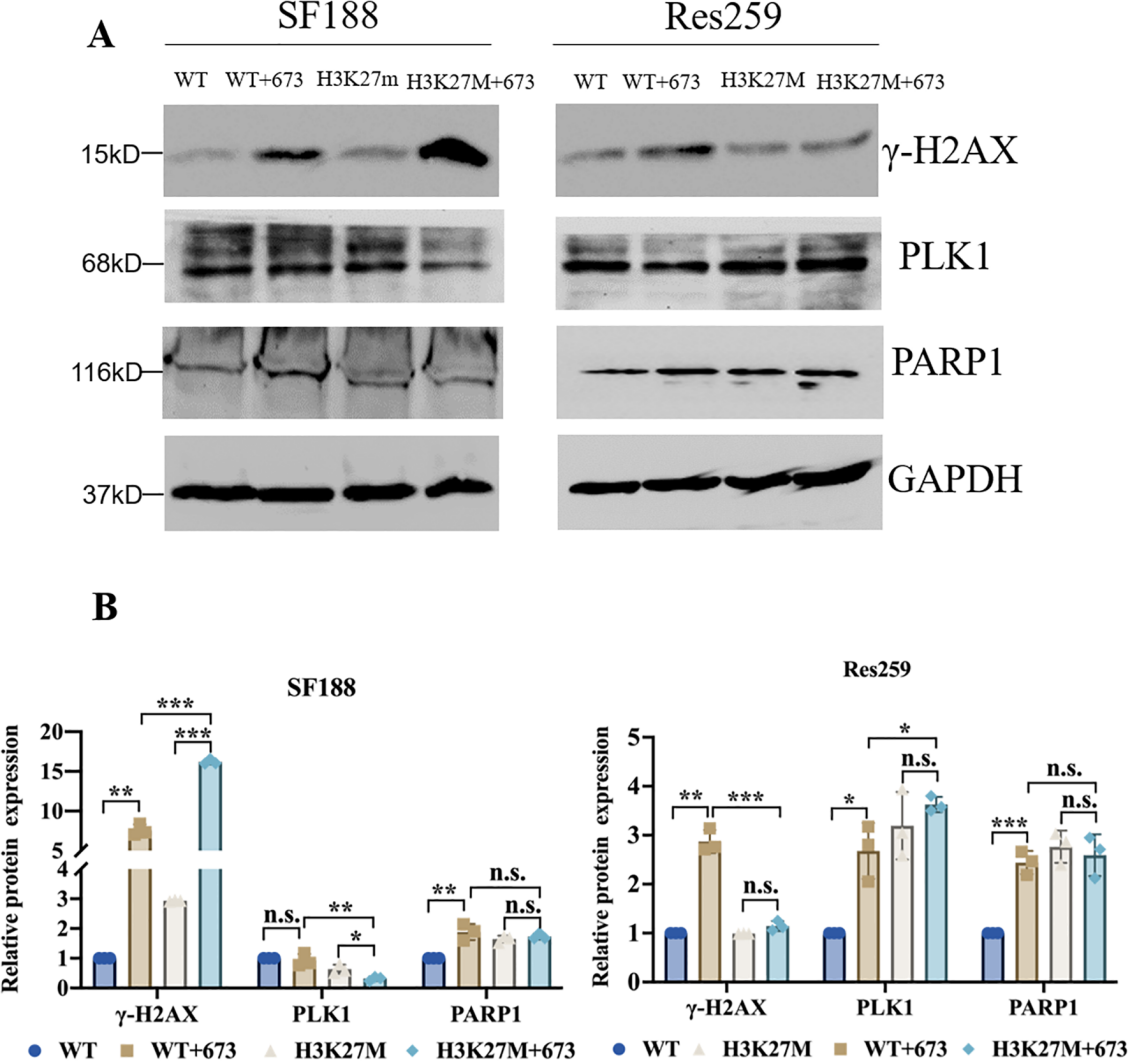
BMN673 treatment induced DNA damage in H3K27M-mutant SF188 cells. **(A)** Western blot analysis of PLK1, PARP1 and γ-H2AX in H3K27M-mutant SF188 and H3K27M-mutant Res259 cells and the corresponding WT cells treated with BMN673. **(B)** Densitometric quantification of the proteins in **(A)**. H3K27M-mutant SF188 cells, WT SF188 cells, H3K27M-mutant Res259 cells and WT Res259 cells were treated with BMN673 for 48 h, GAPDH was used as a loading control. The data are shown as the mean ± SD from three independent experiments. Student’s t test was used to calculate *p* values. *p < 0.05, **p < 0.01, ***p < 0.001 versus the corresponding control.

Having determined that the H3K27M mutation results in an aberrant increase in NHEJ activity in H3K27M-mutant SF188 cells, we next analyzed the DNA repair phenotype of H3K27M-mutant SF188 cells upon BMN673 inhibition. As we all know, γ-H2AX is a known marker of DNA damage. The Western blot results revealed that after treatment with BMN673, the level of γ-H2AX was significantly increased in SF188 cells, especially H3K27M-mutant SF188 cells (**Figures 3A, B**). However, although BMN673 treatment increased the level of γ-H2AX in WT Res259 cells, it did not increase the γ-H2AX level in H3K27M-mutant Res259 cells (**Figures 3A, B**). PLK1 is a master mitotic regulator and controls almost every step of the G2/M phase, including mitotic entry, chromatid segregation and cytokinesis (36). Western blot analysis showed that BMN673 treatment significantly decreased PLK1 expression in H3K27M-mutant SF188 cells compared to that in WT SF188 cells (**Figures 3A, B**). However, BMN673 treatment did not reduce PLK1 expression in H3K27M-mutant Res259 cells (**Figures 3A, B**). Moreover, since PLK1 expression was decreased in H3K27M-mutant SF188 cells after treatment with BMN673, we next assessed the impact of PARP1 inhibition on the cell cycle distribution of H3K27M-mutant SF188 cells. As shown in **Supplementary Figures S4A, B**, treating cells with BMN673 induced G2/M arrest and increased the percentage of tetraploid cells, especially among H3K27M-mutant SF188 cells. Taken together, these results indicate that BMN673 treatment induced obvious DNA damage in H3K27M-mutant SF188 cells.

### Synergy between BMN673 and the H3K27M mutation in SF188 cells results in PARP trapping

**Figure 4:**
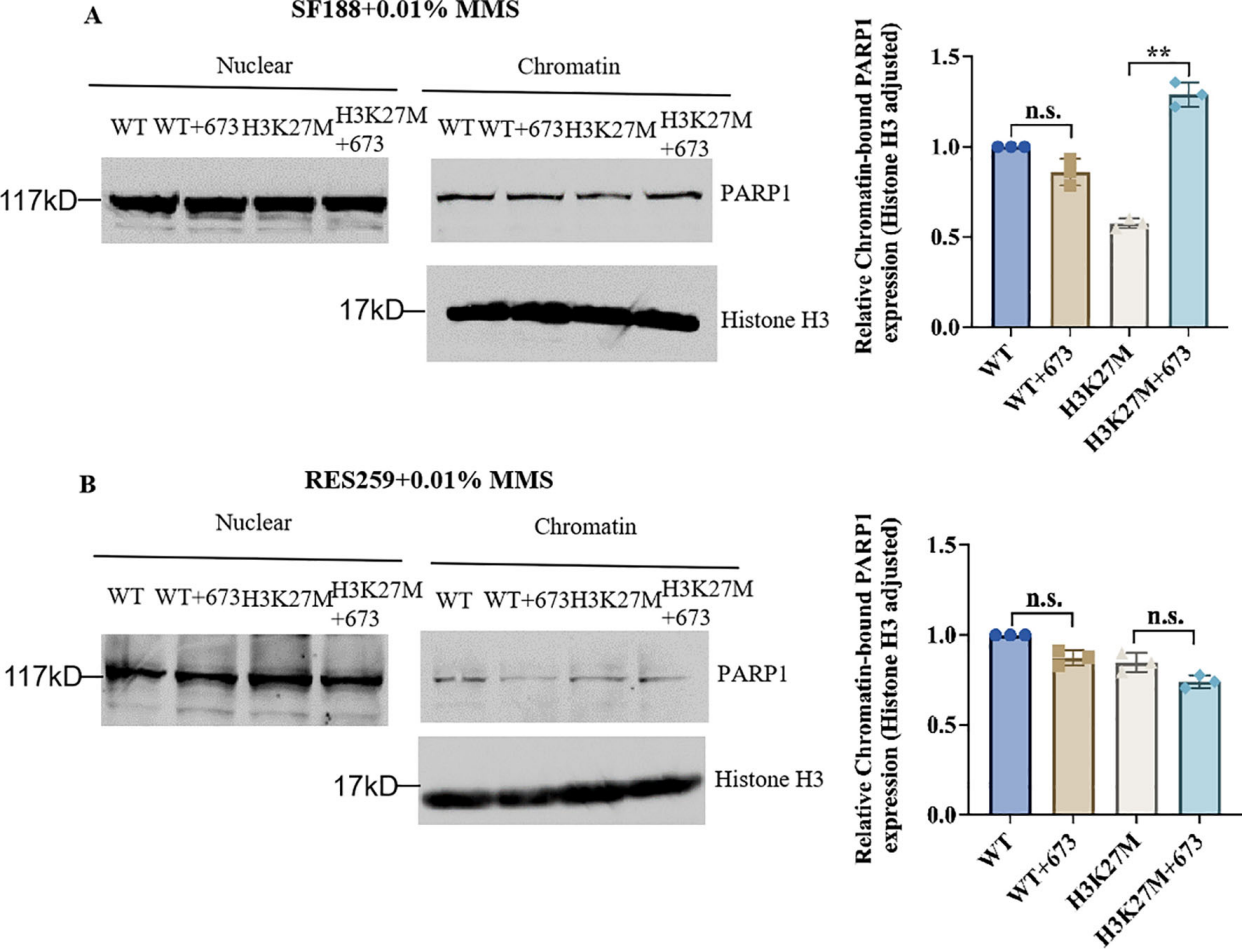
Synergy between BMN673 and the H3K27M mutation results in PARP trapping. **(A)** Left: Western blot analysis of chromatin-bound PARP1 in H3K27M-mutant SF188 cells and WT SF188 cells; Right: Densitometric quantification of chromatin-bound PARP1 normalized to histone H3. **(B)** Left: Western blot analysis of chromatin-bound PARP1 in H3K27M-mutant Res259 and WT Res259 cells. Right: Densitometric quantification of chromatin-bound PARP1 normalized to histone H3. The cells were pretreated with BMN673 (2 μM) for 6 h and then treated with MMS (0.01%) for 2 h Chromatin-bound proteins were extracted and analyzed via Western blot with the indicated antibodies. PARP1 levels were normalized to histone H3 levels. The data are shown as the mean ± SD from three independent experiments. **p < 0.01 versus the corresponding control.

Studies have revealed that PARP inhibitors induce cytotoxicity via two mechanisms, namely, by reducing the catalytic activity of PARP1 and by trapping PARP1. PARP inhibitors trap PARP1 on DNA to form PARP1–DNA complexes, and these complexes influence the process of DNA replication (20, 21, 37). From the results in **Figures 3A, B**, we found that BMN673 treatment did not reduce the protein level of PARP1. Next, to explore the specific therapeutic mechanism of BMN673 in H3K27M-mutant cells, we cotreated the indicated cells with BMN673 and methyl methanesulfonate (MMS; an agent that induces DNA alkylation damage) and subjected them to subcellular fractionation. The results showed that PARP1 trapping on chromatin dramatically increased in H3K27M-mutant SF188 cells compared with WT SF188 cells after treatment with the combination of MMS and BMN673 (**Figure 4A**). However, there was no obvious change in the accumulation of PARP1 in the chromatin-bound fraction of H3K27M-mutant Res259 cells after treatment with BMN673 or MMS (**Figure 4B**). Taken together, these results indicate that PARP1 trapping may be the main mechanism underlying the sensitivity of H3K27M-mutant SF188 cells to BMN673.

### PALB2 deficiency sensitizes H3K27M-mutant Res259 cells to BMN673

**Figure 5:**
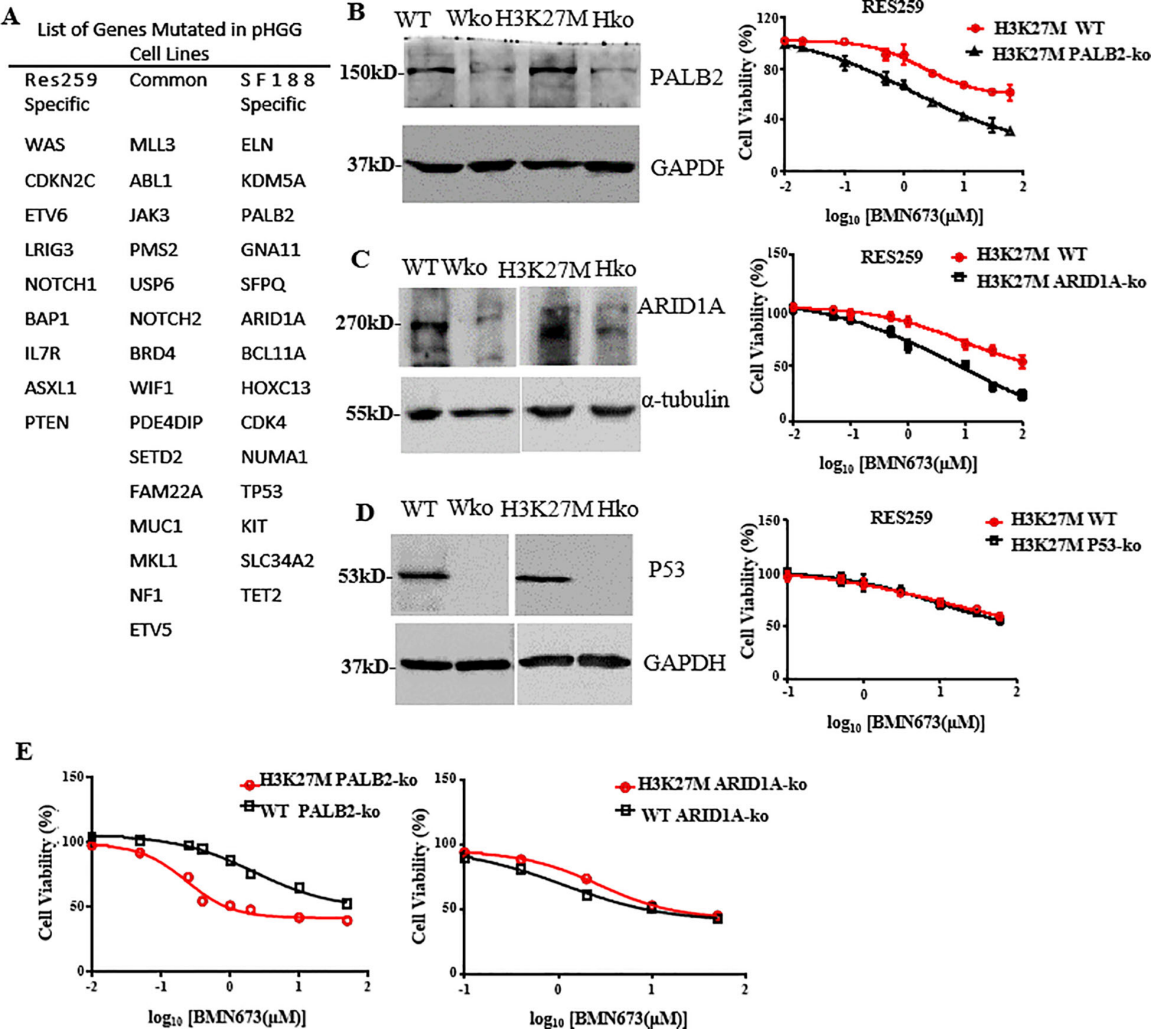
PALB2 deficiency increases the sensitivity of H3K27M-mutant Res259 cells to BMN673. **(A)** List of genes mutated in the Res259 and SF188 cell lines, respectively. **(B)** Left: Western blot analysis showing the expressions of PALB2 in the four indicated cell lines: WT Res259 cells (WT), WT Res259 cells with PALB2 deficiency (Wko), H3K27M-mutant Res259 cells (H3K27M) and H3K27M-mutant Res259 cells with PALB2 deficiency (Hko). Right: Dose–response curves for H3K27M-mutant Res259 cells with PALB2 deficiency and parental H3K27M-mutant Res259 cells treated with BMN673. **(C)** Left: Western blot analysis showing the expressions of ARID1A in the four indicated cell lines. Right: Dose–response curves for H3K27M-mutant Res259 cells with ARID1A deficiency and parental H3K27M-mutant Res259 cells treated with BMN673. **(D)** Left: Western blot analysis showing the expressions of P53 in the four indicated cell lines. Right: Dose–response curves for H3K27M-mutant Res259 cells with P53 deficiency and parental H3K27M-mutant Res259 cells treated with BMN673. **(E)** Left: Dose–response curves for H3K27M-mutant Res259 cells with PALB2 deficiency and WT Res259 cells with PALB2 deficiency treated with BMN673; Right: Dose–response curves for H3K27M-mutant Res259 cells with ARID1A deficiency and WT Res259 cells with ARID1A deficiency treated with BMN673. The data are shown as the mean ± SD from three independent experiments.

The above results demonstrated that BMN673 had different effects on H3K27M-mutant SF188 cells and H3K27M-mutant Res259 cells. According to the concept of synthetic lethality, we hypothesized that the combination of other gene mutations with the H3K27M mutation could further increase the sensitivity of cells to BMN673. A whole-exome sequencing analysis of pHGGs conducted by Mackay et al. (38) revealed several gene mutations with specificity for the parental SF188 or Res259 cells (**Figure 5A**). Notably, among these mutated genes, PALB2, ARID1A and P53, which are related to DNA damage and tumorigenesis, were mutated only in SF188 cells. To determine which of these proteins is responsible for sensitivity to BMN673, the established H3K27M-mutant Res259 cells or WT Res259 cells with stable deficiency of ARID1A, P53 or PALB2 were treated with the indicated concentrations of BMN673; the results revealed that BMN673 had substantially greater potency against H3K27M-mutant Res259 cells with ARID1A or PALB2 deficiency than against parental H3K27M-mutant Res259 cells, whereas BMN673 treatment had no effect on H3K27M-mutant Res259 cells with P53 deficiency (**Figures 5B–D**). Moreover, as shown in **Figure 5E**, our results further revealed that H3K27M-mutant Res259 cells with PALB2 deficiency were more sensitive to BMN673 than WT Res259 cells with PALB2 deficiency, whereas no significant difference was detected between H3K27M-mutant Res259 cells with ARID1A deficiency and WT Res259 cells with ARID1A deficiency (**Figure 5E**). These results together suggest that PALB2 deficiency may be the main contributor to the sensitivity of H3K27M-mutant SF188 cells to BMN673 and that PALB2 deficiency can increase the sensitivity of H3K27M-mutant Res259 cells.

### BMN673 treatment decreased the level of PLK1 and increased the level of γ-H2AX as well as the amount of PARP1 trapped on chromatin in H3K27M-mutant Res259 cells with PALB2 deficiency

**Figure 6:**
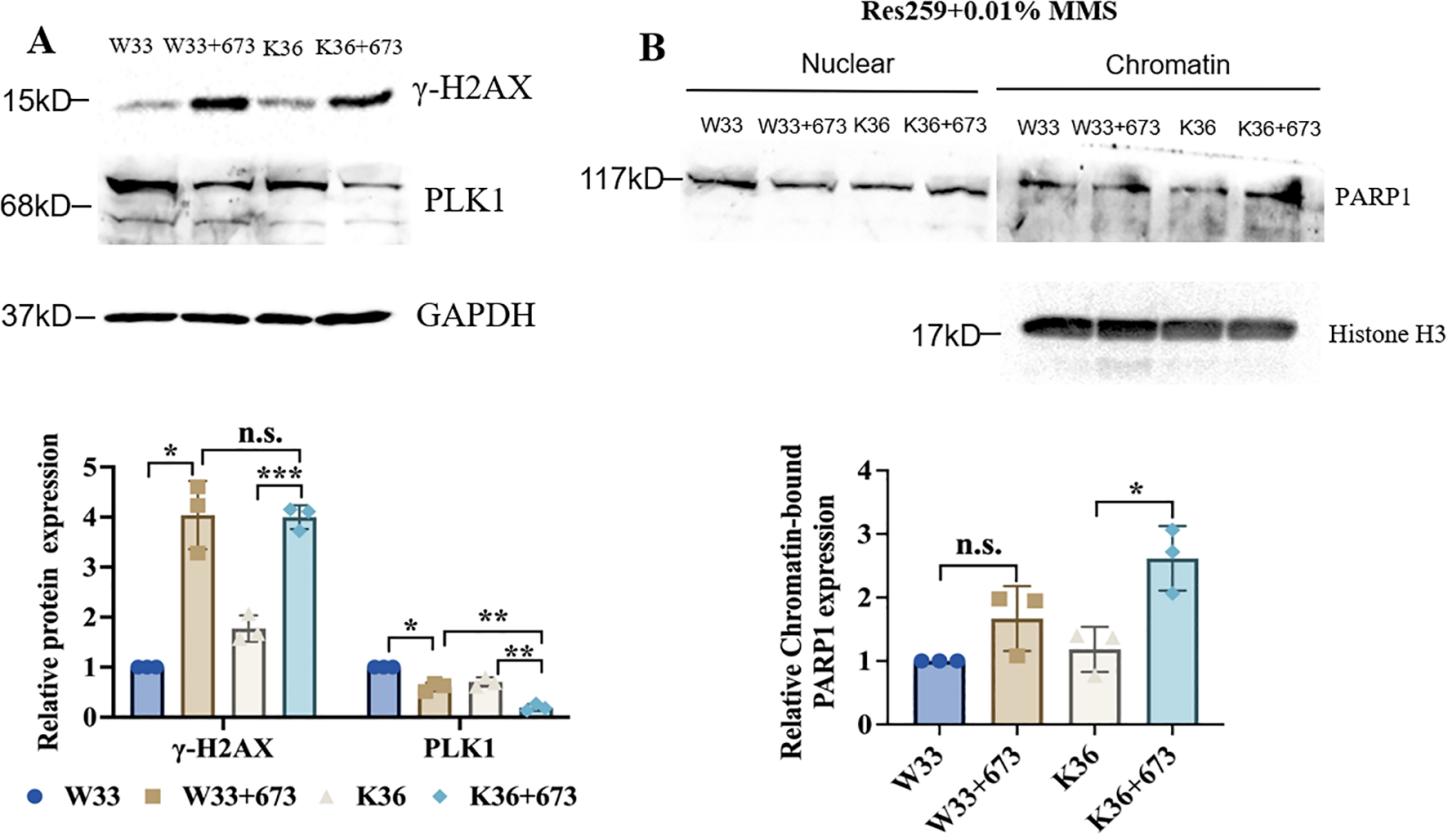
BMN673 treatment decreased the level of PLK1 and increased the level of γ-H2AX as well as the amount of PARP1 trapped on chromatin in H3K27M-mutant-PALB2-KO Res259 cells. **(A)** Upper panel: Western blot analysis of PLK1 and γ-H2AX in H3K27M-mutant-PALB2-KO Res259 cells (K36) and the corresponding WT cells (W33) treated with BMN673. Lower panel: Densitometric quantification of the proteins. **(B)** Upper panel: Western blot analysis of chromatin-bound PARP1 in H3K27M-mutant-PALB2-KO Res259 (K36) and H3 WT-PALB2-KO Res259 cells (W33). Lower panel: Densitometric quantification of chromatin-bound PARP1 normalized to histone H3. The data are shown as the mean ± SD from three independent experiments. *p < 0.05, **p < 0.01, ^***^ *p* < 0.001 versus the corresponding control.

To further explore the connection between PALB2 to PARP sensitivity, based on the H3K27M mutant and WT Res259 cells that we constructed, we subsequently knocked out PALB2 via CRISPR/Cas9 technology and generated stable PALB2-KO-H3K27M-mutant Res259 cells (K36) and PALB2-KO-H3 WT Res259 cells (W33). We then performed Western blot analysis, and the results, as illustrated in **Figure 6A**, revealed that the γ-H2AX expression level in K36 cells significantly increased after BMN673 treatment, whereas the protein level of PLK1 significantly decreased in K36 cells after BMN673 treatment, indicating that BMN673 treatment may also induce DNA damage when PALB2 is knocked out in H3K27M-mutant Res259 cells. Furthermore, through a PARP trapping experiment (**Figure 6B**), the results revealed that the amount of PARP1 in chromatin also increased when PALB2 was knocked out in H3K27M-mutant Res259 cells. These results further reveal the close correlation between PALB2 deficiency, BMN673 and H3K27M mutation.

## Discussion

pHGG is a deadly brain tumor type with a two-year survival rate of less than ten percent after diagnosis (39). One important feature of pHGG is heterozygous, dominant point mutations in the H3F3A gene, which encodes the H3 histone variant (H3K27M) (40). While clinical trials of diverse therapeutic modalities, including small molecule inhibitors, immunotherapy, and convection-enhanced delivery, are underway, no treatment has been shown to improve overall or progression-free survival in patients with pHGG (2, 41). Due to the extremely poor prognosis and lack of effective therapies for pHGG, more potential approaches for its treatment are needed.

For this study, we focused on exploring more potential drugs that target H3K27M-mutant pHGG cells. First, pairwise dose–response screening of a drug library with two pHGG cell lines generated in our lab that harbor the H3K27M mutation (H3K27M-mutant SF188 cells and H3K27M-mutant Res259 cells) revealed the BMN673, JQ1 and AZ960 as potential drugs targeting H3K27M-mutant pHGG cells; in further validation experiments, the BMN673 unexpectedly showed considerable and potentially cytotoxic effects against H3K27M-mutant SF188 cells. However, BMN673 did not have any effect on H3K27M-mutant Res259 cells.

Several small-molecule compounds targeting the oncogenic H3K27M mutation have been reported to inhibit the growth of glioblastoma *in vitro* and *in vivo*. For example, the histone deacetylase inhibitor panobinostat has demonstrated therapeutic efficacy both *in vitro* and in orthotopic xenograft models of diffuse intrinsic pontine glioma (DIPG) (26). Another study reported that EZH2 inhibitors had obvious effects on 6 pHGG cell lines as well as a pediatric HGG cell line with the G34R mutation in histone H3 (15). Moreover, similar antiproliferative effects were observed in pHGG cells with the inhibition of EED and SUZ12, both of which are PRC2 subunits, in a study performed by Piunti et al. (42). Balakrishnan et al. reported that BMI1 constitutes a major epigenetic vulnerability in H3K27M-DIPG cells and that the combination of the BMI1 inhibitor PTC028 with the senolytic drug obatoclax is a promising therapeutic strategy for DIPG (13). Given that these inhibitors have been tested only in preclinical models, clinical trials need to be conducted to determine their efficacy in pHGG patients. ONC201 has shown promise in the clinical setting as an antitumor agent with high central nervous system (CNS) penetration and is currently being investigated in clinical trials for adults and pediatric glioblastoma (14). However, the toxicity of existing therapies and the emergence of resistance hinder the efficacy of current therapeutic protocols, and none of the potential targeted drugs are currently in clinical use (43). Therefore, finding alternative targeted therapeutic strategies for H3.3 or H3.1 variant mutant histone H3K27M mutant pHGG remains a major research challenge.

Here, we showed that the PARP1 inhibitor BMN673 had a considerable potential selective cytotoxic effect on H3K27M-mutant SF188 cells. PARP1 is a well-known component of the DNA damage response. Several PARP1 inhibitors, such as olaparib, niraparib, and BMN673, have been developed. Chemical inhibition of PARP1 has been demonstrated to decrease the proliferation and metastatic capacity of several types of tumor cells (44, 45). The results of clinical trials exploring synthetic lethality using PARP inhibitors as a treatment for platinum-sensitive BRCA1/2-mutated breast and ovarian tumors are promising (20). Moreover, glioblastoma have been shown to be therapeutically vulnerable to PARP1 inhibition, and olaparib effectively increased the radiosensitivity of glioblastoma cells *in vitro* (46). However, to date, no data are available on the effect of BMN673 on pHGG, especially pHGG with the H3K27M mutation.

Recent studies have revealed that the trapping of PARP1 onto damaged chromatin is central to the synergistic cytotoxicity observed when PARP inhibitors are combined with alkylating agents and that PARP inhibition results in the accumulation of recombinogenic substrates marked by γ-H2AX nuclear foci (21, 22). In the present study, it was found that the level of γ-H2AX, a DNA damage marker, and the abundance of the PARP1 protein in the chromatin-bound fraction of H3K27M-mutant SF188 cells were upregulated after BMN673 treatment, indicating that BMN673 can trap PARP1 on DNA to form PARP1–DNA complexes in H3K27M-mutant SF188 cells. However, BMN673 treatment did not cause obvious PARP1 trapping in H3K27M-mutant Res259 cells, which may be the reason for the resistance of H3K27M-mutant Res259 cells to BMN673. As previous studies noted, in pHGG cells, H3 mutations also promote genome instability, which is one of the major drivers of cellular transformation, and the K27M mutation alters the association of H3 with several DNA repair factors in human cells (47–49). Specially, a study reported that the combination of EZH2 inhibitors and PARP1 inhibitors enhanced lethality in homologous recombination (HR)-proficient and CARM1-high ovarian cancer (50). In addition, Giacomini et al. reported that the H3K27M mutation could drive aberrant repair of replication-associated damage via nonhomologous end joining (NHEJ) (33). Another report demonstrated that shieldin complexes are key regulators of NHEJ and that the deletion of shieldin endows BRAC1-deficient cells with resistance to PARP1 inhibitors (34). Consistently, the results of our study also revealed that the H3K27M mutation significantly increased NHEJ repair efficiency in H3K27M-mutant SF188 cells, indicating the important role of NHEJ activity in the effect of BMN673 to H3K27M-mutant SF188 cells.

Notably, our study showed that the effect of BMN673 on H3K27M-mutant Res259 cells were directly opposite those on H3K27M-mutant SF188 cells, prompting us to explore the reasons. As mentioned in previous studies (51), the discovery of the high frequency of H3K27M mutations in pHGG indicates that this mutation can also be exploited therapeutically on the basis of a concept called synthetic lethality, which refers to a genetic interaction between two or more genes where deficiency of either gene alone does not affect cell viability, but combined deficiency of both genes results in lethality. Therefore, according to the concept of synthetic lethality and the results of whole-exome sequencing of the two parental pHGG cell lines (SF188 and Res259), we found that ARID1A, PALB2 and TP53, which are important for DNA repair, were mutated only in SF188 cells. ARID1A is a subunit of the evolutionarily conserved SWI/SNF chromatin remodeling complex, which modulates DNA accessibility to components involved in cellular processes related to chromatin structure, such as transcription, DNA replication, and DNA repair (52). PALB2 plays a crucial role in genome stability and the DNA repair process (53). It has been identified as a major BRCA2 binding partner that is required for the stability, chromatin association and HR function of BRCA2 (54). The transcription factor TP53 functions as a central hub that translates various stress signals into diverse cellular processes or fates, such as DNA repair, cell cycle arrest, and cell death (55, 56). On the basis of these published observations, we hypothesized that the mutation of these three genes may exert a synthetic lethality effect with BMN673 treatment in H3K27M-mutant SF188 cells. Intriguingly, BMN673 was found to exhibit substantially greater potency against H3K27M-mutant Res259 cells with ARID1A deficiency or PALB2 deficiency than against the corresponding H3K27M-mutant Res259 cells. However, H3K27M-mutant Res259 cells with P53 deficiency remained resistant to BMN673. In addition, our results revealed that only PALB2 deficiency increased the sensitivity of WT Res259 cells to BMN673 treatment, which suggested that PALB2 deficiency can increase the sensitivity of H3K27M-mutant Res259 cells to treatment with BMN673. Notably, previously studies showed that the H3K27M mutation could also reduce RAD51 and FANCD2 focus formation (impairing HR), while simultaneously increasing 53BP1 foci (promoting aberrant NHEJ) (33, 57). Hence, the combined effects of H3K27M-mediated HR deficiency and PALB2 loss likely underlie the enhanced synthetic lethality observed with BMN673, establishing a more vulnerable “BRCAness” state. It should be noted that SF188 has been reported to have high levels of MYC, suggesting that the high level of MYC may be also one reason for the increased sensitivity of SF188 to BMN673. On the basis of the literature (56, 58, 59), BMN673 treatment may inhibit the expression of MYC, which could be one of the reasons why BMN673 is sensitive to SF188 cells. As Res259 cells do not inherently have high MYC expression, resulting in lower sensitivity of Res259 cells to BMN673. However, this hypothesis needs to be further explored.

However, it is indeed possible that the effects observed are specific to BMN673. We must acknowledge that this efficacy may not be universally present. It may not be effective for all H3K27M-mutant high-grade pediatric glioma cells. We will conduct more in-depth studies to determine in which specific types of H3K27M mutant gliomas BMN673 might be effective. Additionally, this study also has another several limitations. Firstly, only the SF188 and Res259 cell lines were used as experimental materials for constructing the H3K27M overexpression mutant cell lines. This approach may somewhat weaken the accuracy and general applicability of the conclusions. Although using two cell lines for the H3K27M overexpression mutant study may have some exploratory value, it is essential to use animal models and primary H3WT/K27M cells to fully assess the effectiveness of BMN673 in treating H3K27M-mutant pediatric gliomas. We will thus establish orthotopic xenograft models of H3K27M-mutant pediatric gliomas or actively obtain primary cells from clinical tissues to further examine the efficacy of BMN673. A second limitation of this study is that the underlying mechanism was not further evaluated. We will use multidisciplinary and integrative approaches (such as rescue experiments) to gain mechanistic insights into the specific regulation between H3K27M mutation, PALB2 and PARP inhibition. More important, The mechanisms by which additional changes in the genetic makeup result in synthetic lethality with BMN673 in H3K27M gliomas will be further investigated. We hypothesize that the H3K27M mutation causes widespread changes in gene expression. These changes in gene expression in H3K27M-mutant gliomas and mutations in genes such as ARID1A or PALB2 may cooperate to sensitize H3K27M-mutant cancers to BMN673. Thirdly, although another study showed that H3K27M-mutant cells exhibited sensitivity to another PARP1 inhibitor-olaparib treatment (33), to ensure the convince of our conclusions, we did not extend our findings to other PARP inhibitors. In follow-up studies, we will further investigate the effects of other PARP1 inhibitors in the context of our research and plan to validate and extend our current findings using natural H3K27M-mutant glioma models.

## Conclusion

Our study demonstrated that treatment with BMN673 resulted in cell cycle arrest and impaired DSB repair, as well as increased PARP1 trapping and the efficiency of NHEJ repair in SF188 cells harboring the H3K27M mutation, while not in RES259 cells with the same mutation. Furthermore, we found that PALB2 deficiency enhanced the sensitivity of H3K27M-mutant Res259 cells to BMN673, which was originally resistant to BMN673, suggesting that PALB2 mutation might be one reason for the sensitivity of BMN673 drug to H3K27M-mutant SF188 cell line. Taken together, our preliminary findings suggest that the treatment with the BMN673 may be a promising strategy for targeting the H3K27M mutation in pHGG.

## Data Availability

The original contributions presented in the study are included in the article/**Supplementary Material**. Further inquiries can be directed to the corresponding author/s.

## Supplementary material

Supplementary Figure 1 The flowchart for constructing H3 WT and K27M mutant stable cell lines.

Supplementary Figure 2 Dose–response curves for H3K27M-mutant SF188 cells, H3K27M-mutant Res259 cells, WT SF188 cells and WT Res259 cells treated with JQ1 **(A)** or AZ960 **(B)**. The IC50 values in Res259 cells (middle panel) and SF188 cells (right panel) expressing mutated H3K27M are plotted against the IC50 values in the corresponding cells expressing H3 WT.

Supplementary Figure 3 Dose–response curves of H3K27M-mutant SF188 cells, WT SF188 cells, H3K27M-mutant Res259 cells and WT Res259 cells treated with reported inhibitors, including EPZ6438 **(A)**, sorafenib **(B)**, or LBH589 **(C)**. The IC50 values of RES259 (middle) and SF188 (right) cells harboring the H3K27M mutation are plotted against the IC50 values of cells harboring H3 WT.

Supplementary Figure 4 BMN673 treatment induced cell cycle arrest in H3K27M-mutant SF188 cells. **(A, B)** Cell cycle analysis of H3K27M-mutant SF188 cells and WT SF188 cells treated with BMN673. All the cells were treated with 2 μM BMN673 for 48 h, GAPDH was used as a loading control, and the experiments were repeated three times.

Supplementary Table 1 List of mutated genes in SF188 cells.

Supplementary Table 2 List of mutated genes in Res 259 cells.
