# Supplementary figures and images for: PALB2 deficiency may sensitize H3K27M-mutant pediatric HGG cells to BMN673/talazoparib
Source: Front Oncol. 2025 Jun 30;15:1589396. doi: 10.3389/fonc.2025.1589396 (PMC12256224; doi:10.3389/fonc.2025.1589396)

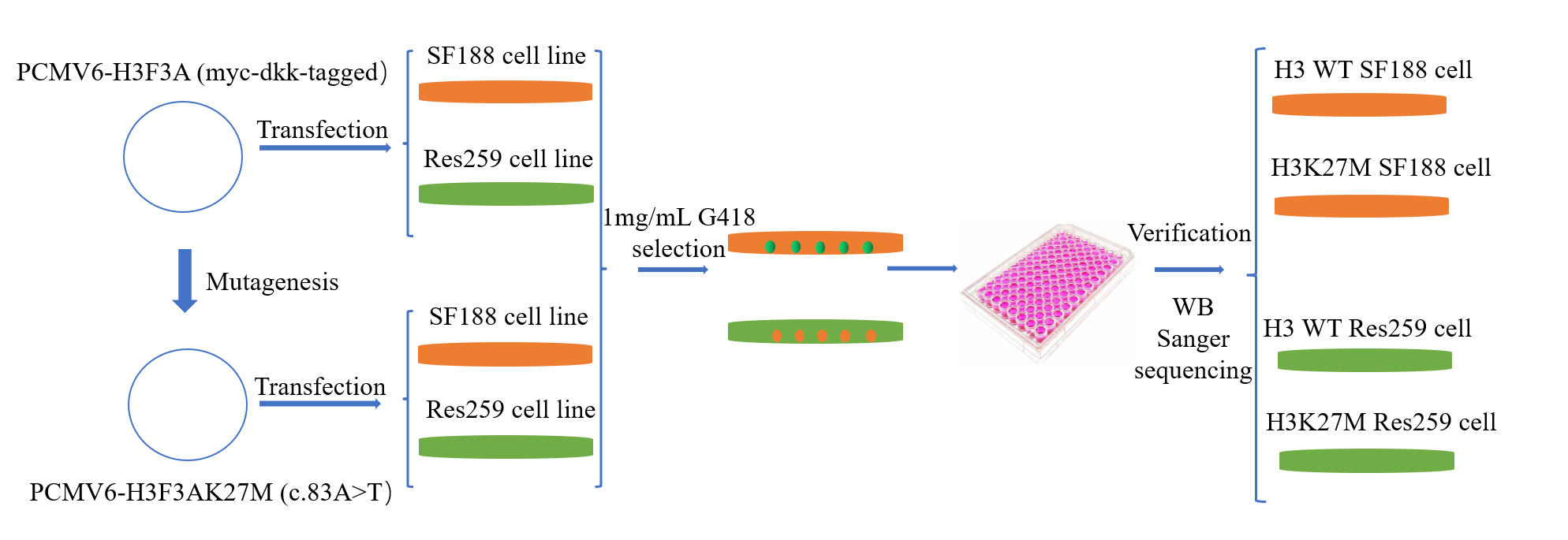

Supplement: Supplementary Figure 1 — The flowchart for constructing H3 WT and K27M mutant stable cell lines. [file Image1.tif]

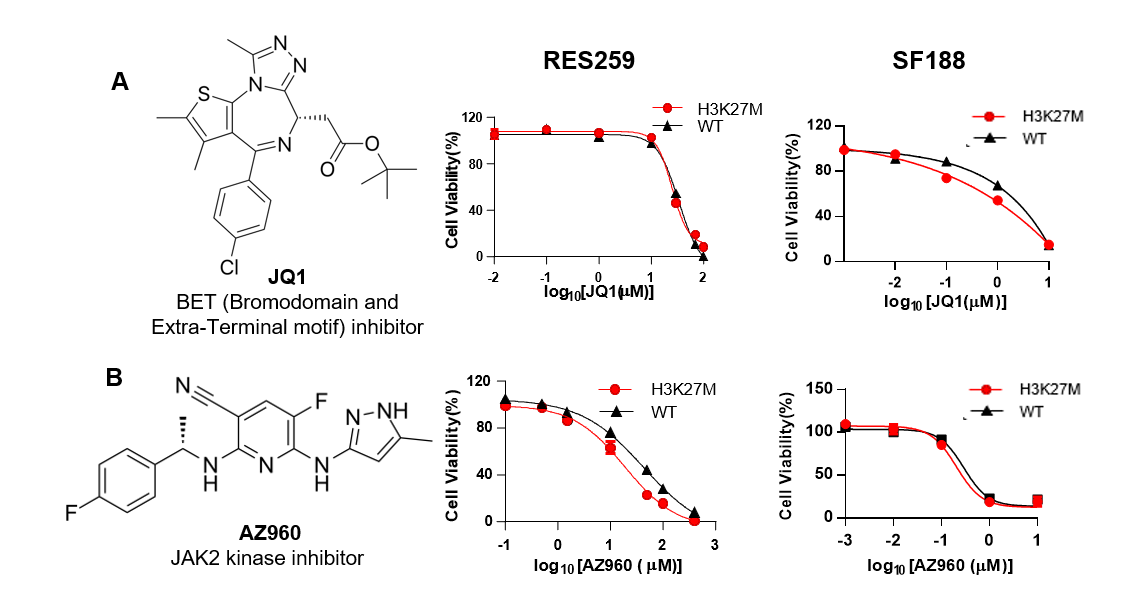

Supplement: Supplementary Figure 2 — Dose–response curves for H3K27M-mutant SF188 cells, H3K27M-mutant Res259 cells, WT SF188 cells and WT Res259 cells treated with JQ1 (A) or AZ960 (B). The IC50 values in Res259 cells (middle panel) and SF188 cells (right panel) expressing mutated H3K27M are plotted against the IC50 values in the corresponding cells expressing H3 WT. [file Image2.tif]

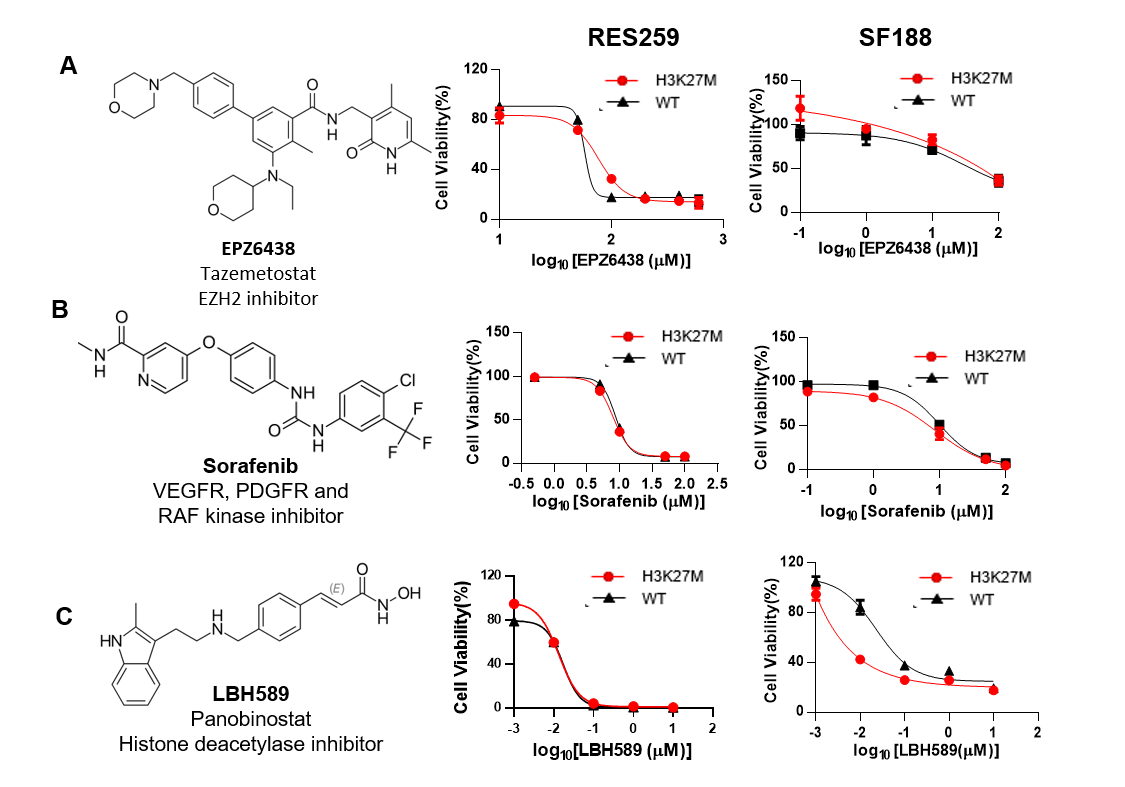

Supplement: Supplementary Figure 3 — Dose–response curves of H3K27M-mutant SF188 cells, WT SF188 cells, H3K27M-mutant Res259 cells and WT Res259 cells treated with reported inhibitors, including EPZ6438 (A), sorafenib (B), or LBH589 (C). The IC50 values of RES259 (middle) and SF188 (right) cells harboring the H3K27M mutation are plotted against the IC50 values of cells harboring H3 WT. [file Image3.tif]

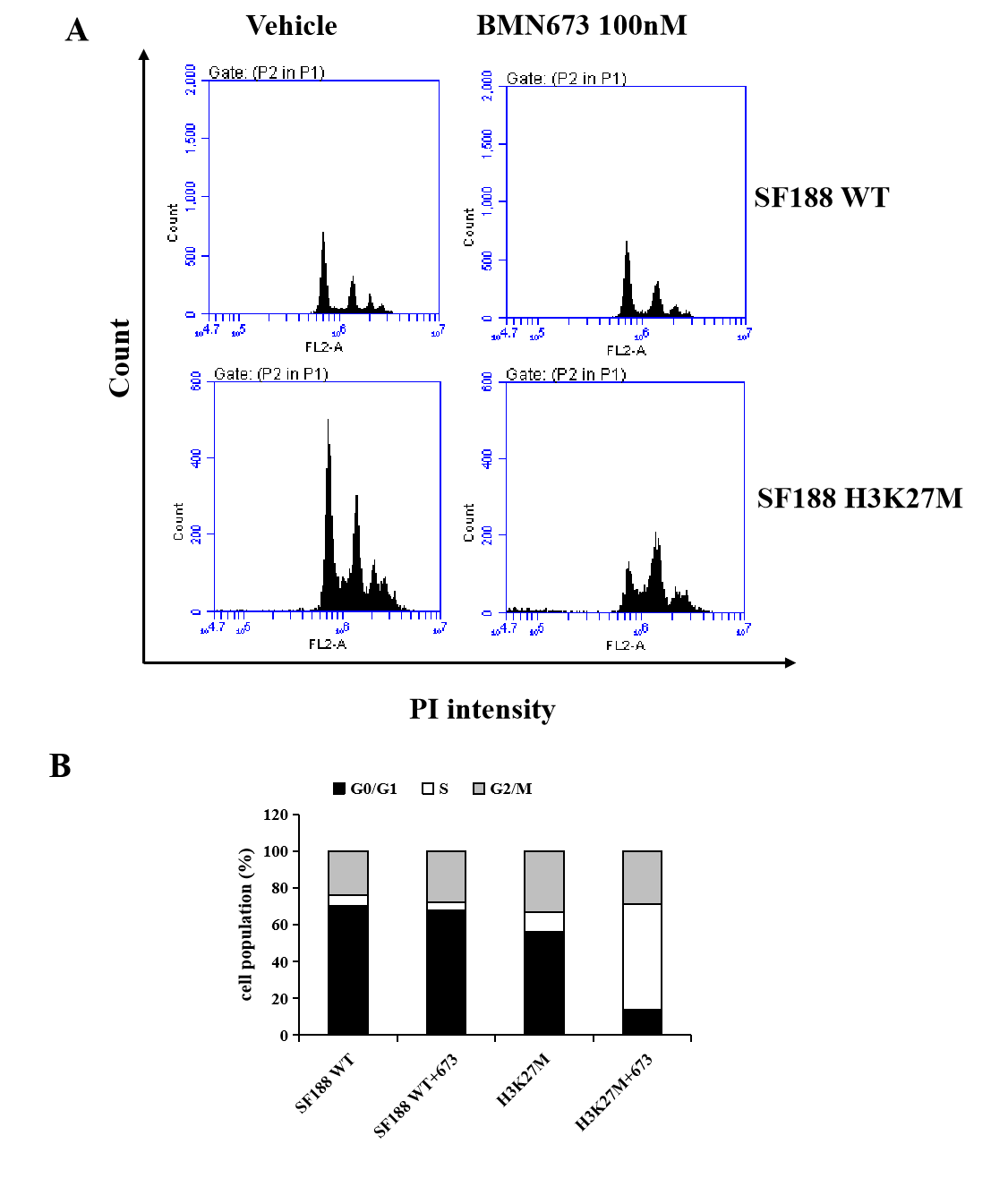

Supplement: Supplementary Figure 4 — BMN673 treatment induced cell cycle arrest in H3K27M-mutant SF188 cells. (A, B) Cell cycle analysis of H3K27M-mutant SF188 cells and WT SF188 cells treated with BMN673. All the cells were treated with 2 μM BMN673 for 48 h, GAPDH was used as a loading control, and the experiments were repeated three times. [file Image4.tif]
