# Supplementary material for: PALB2 deficiency may sensitize H3K27M-mutant pediatric HGG cells to BMN673/talazoparib
Source: Front Oncol. 2025 Jun 30;15:1589396. doi: 10.3389/fonc.2025.1589396 (PMC12256224; doi:10.3389/fonc.2025.1589396)

All the target bands are within the red dashed box.

Fig 1A

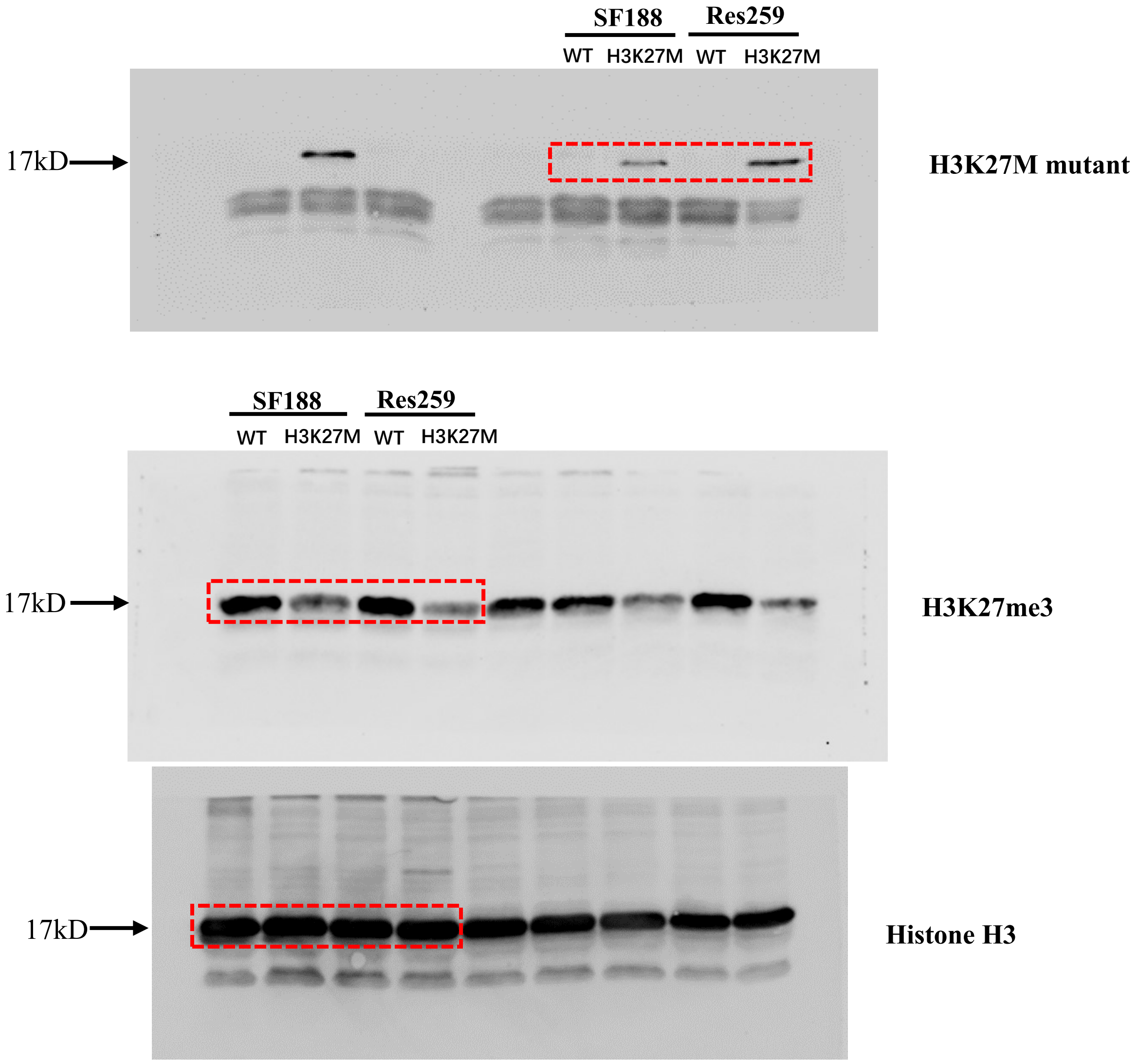

Fig 3A
First time

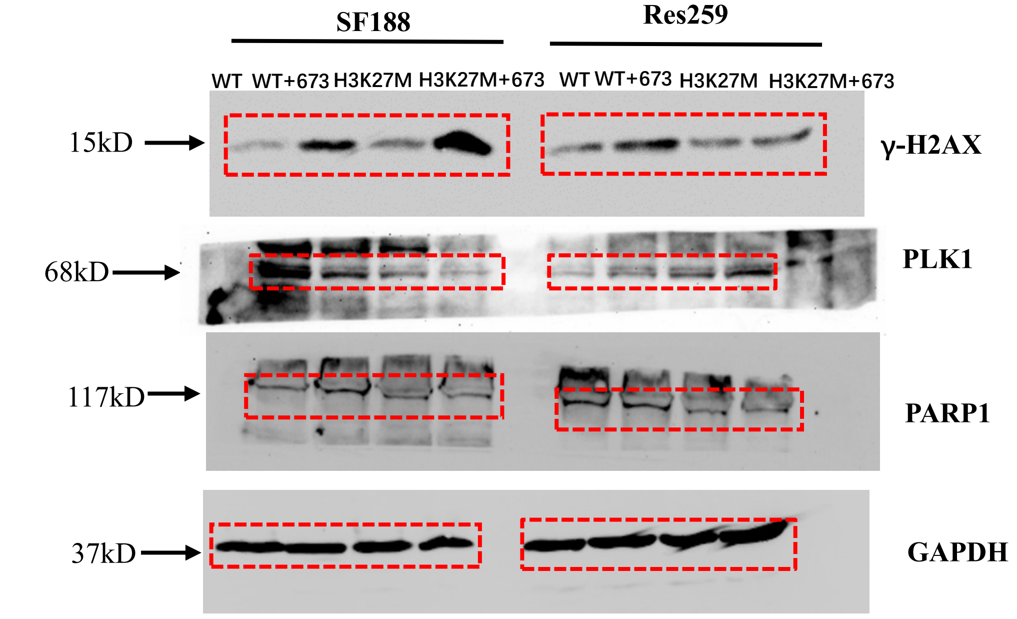

Second time:

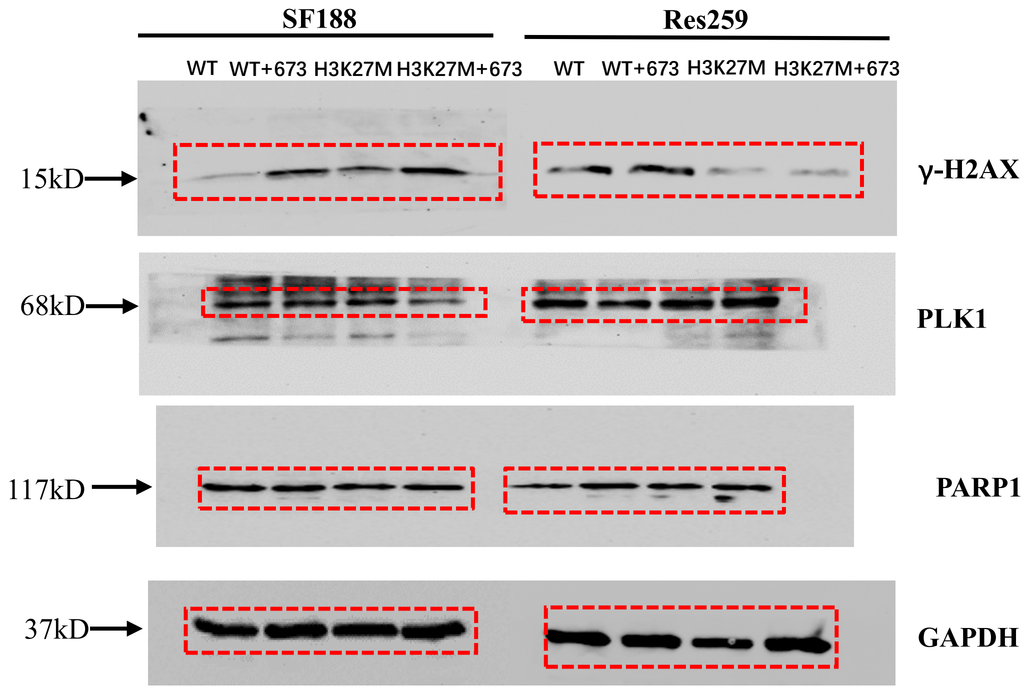

Fig 4A

First time

(SF188 cells+0.01% MMS)

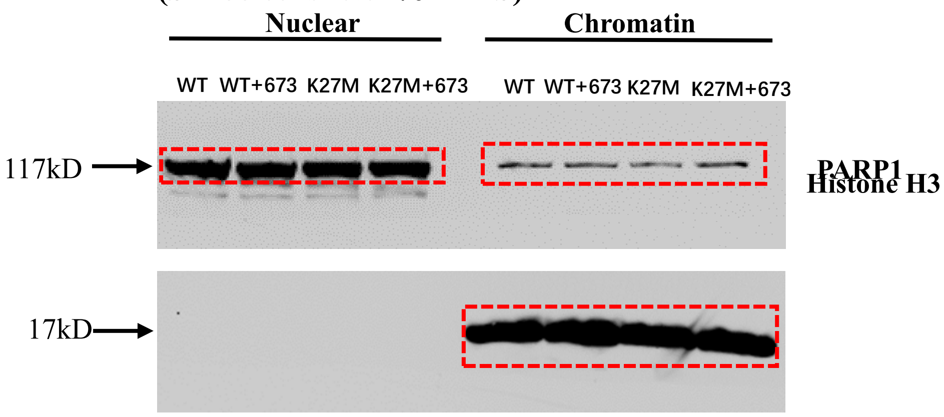

Second time

(SF188 cells+0.01% MMS)

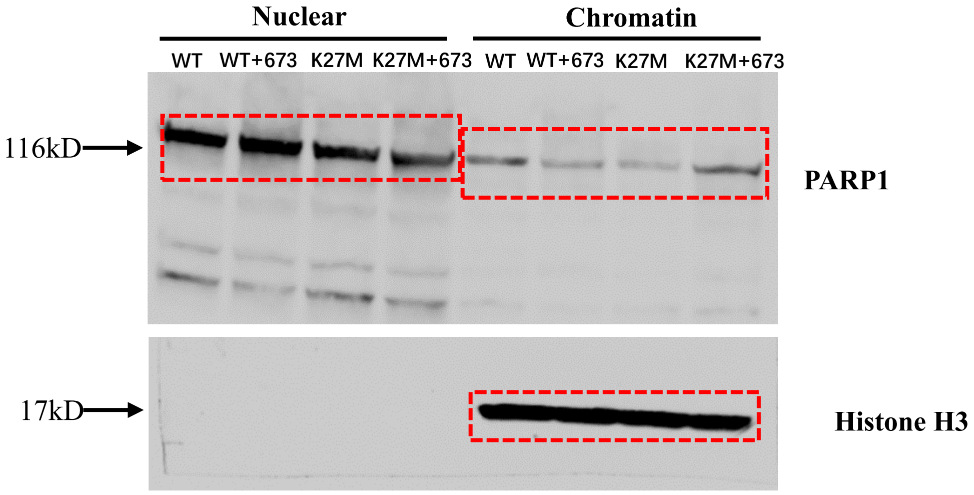

Third time

(SF188 cells+0.01% MMS)

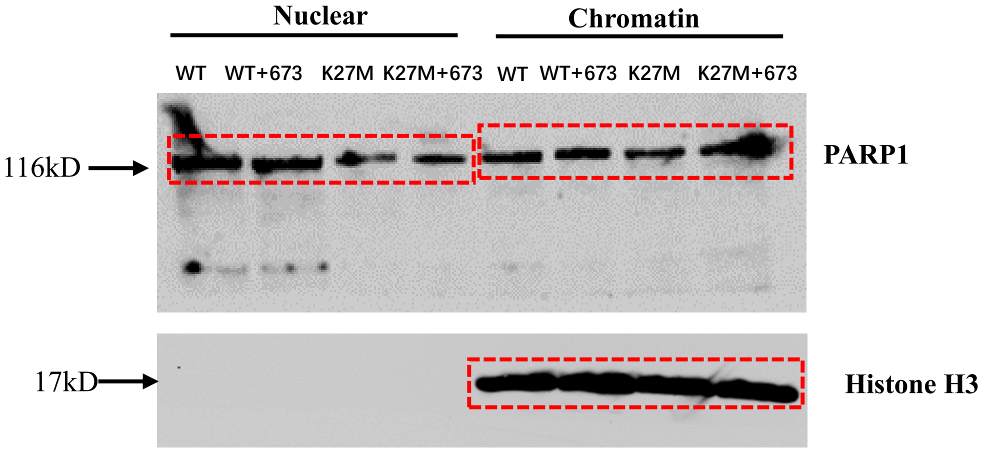

Fig 4B

First time

(Res259 cells+0.01% MMS)

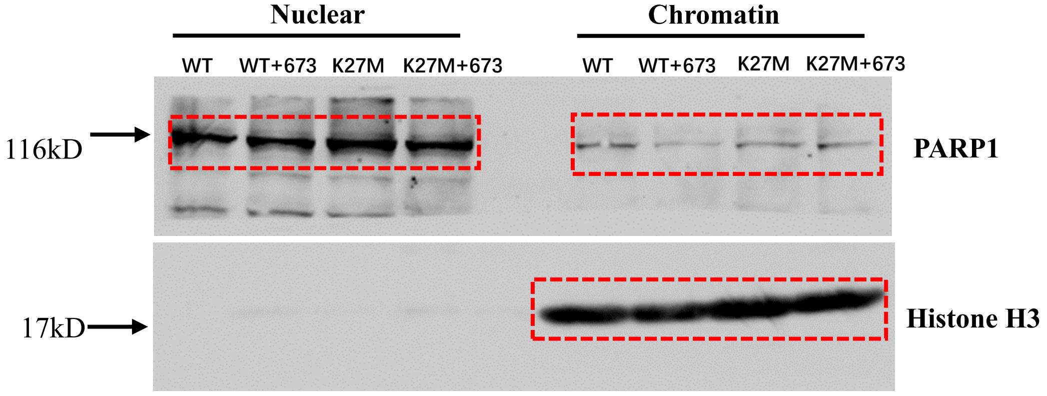

Second time

(Res259 cells+0.01% MMS)

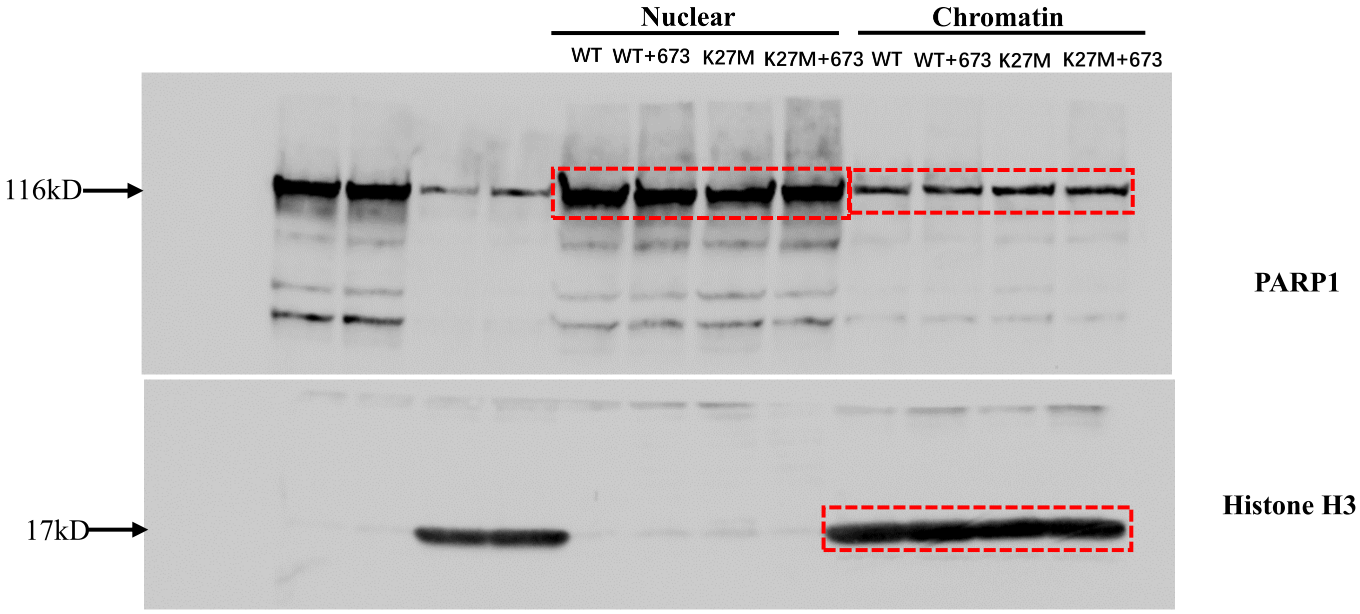

Third time

(Res259 cells+0.01% MMS)

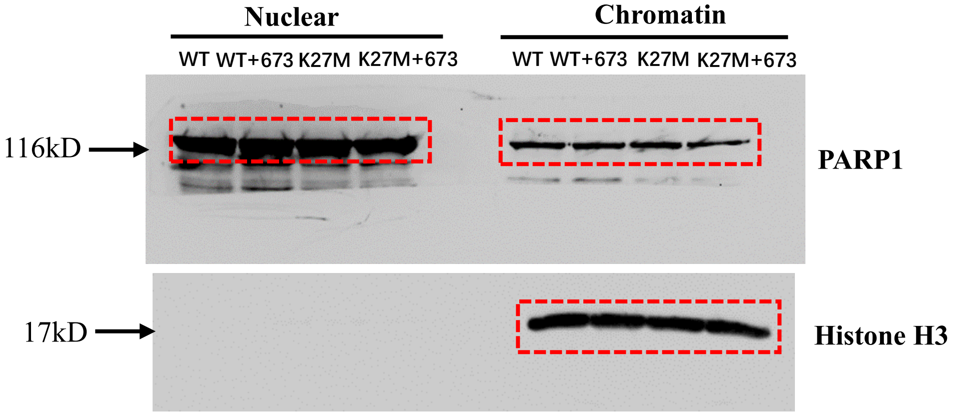

Fig 6B

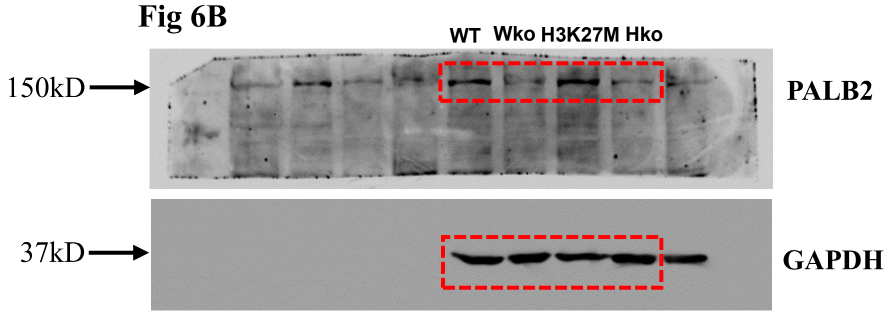

Fig 6C

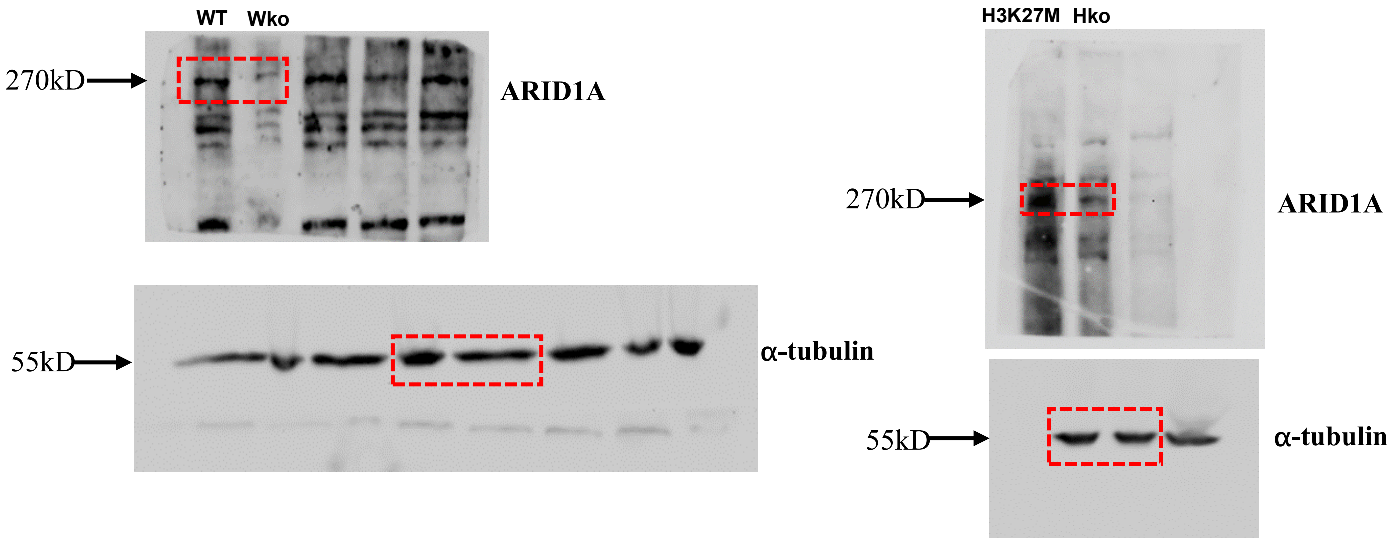

Fig.6D

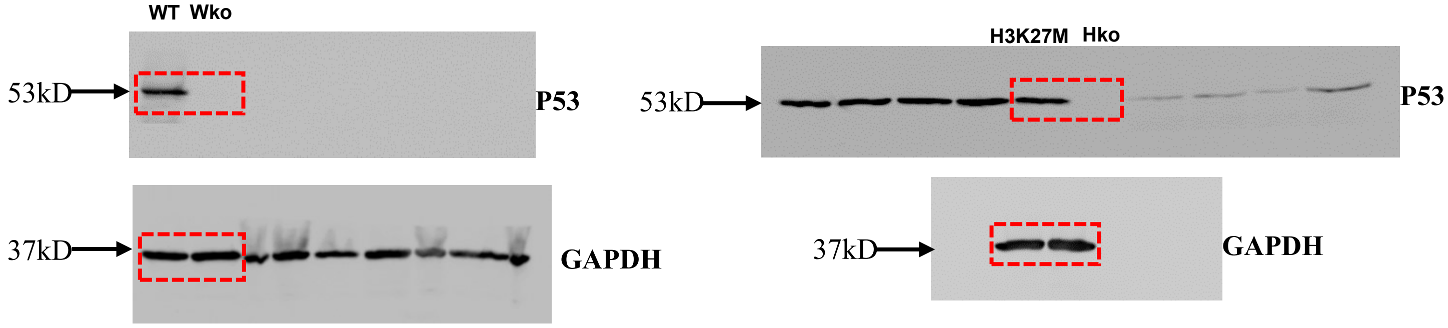

Fig.7A (PALB2-KO-WT Res259 and PALB2-KO-H3K27M-mutant Res259 cells)

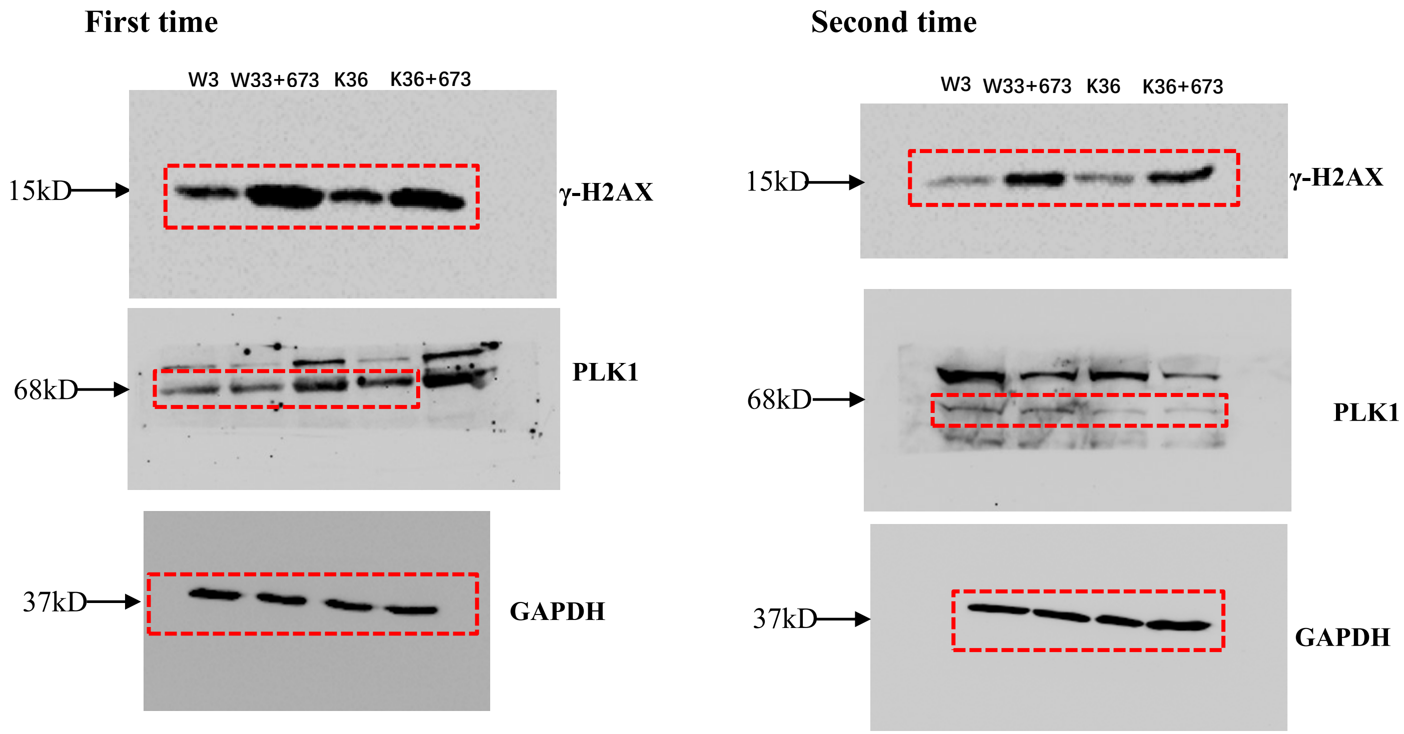

Fig. 7B (PALB2-KO-WT Res259 and PALB2-KO-H3K27M-mutant Res259 cells)
First time

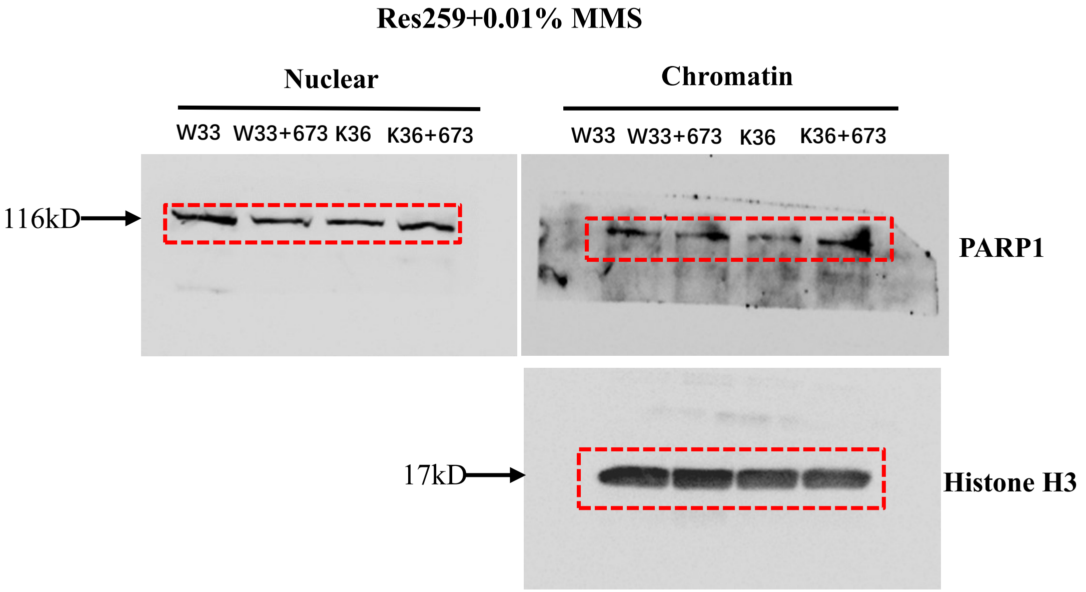

Second time:

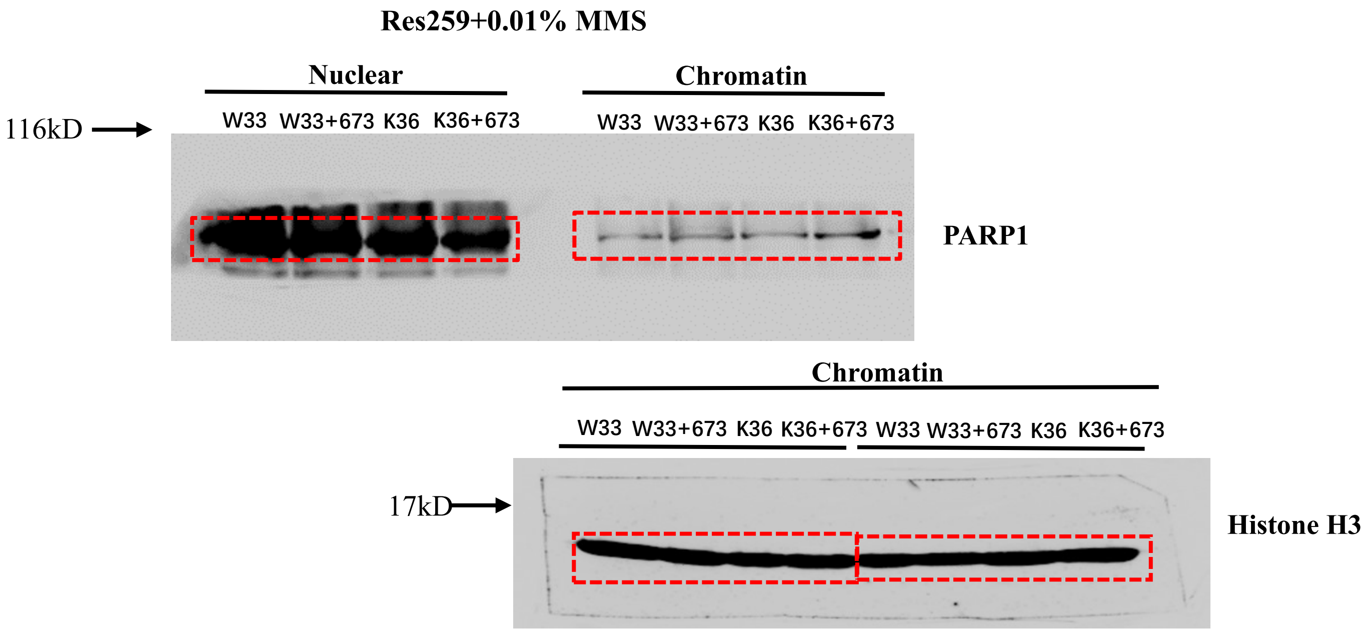

Supplement: Supplementary file 7 [file DataSheet1.pdf]
